# Glycans Modulate the Adsorption of RBD Glycoproteins on Polarizable Surfaces

**DOI:** 10.1021/acs.jcim.5c02363

**Published:** 2026-01-15

**Authors:** Antonio M. Bosch-Fernández, Willy Menacho, Rubén Pérez, Horacio V. Guzman

**Affiliations:** † Departamento de Física Teórica de la Materia Condensada, 16722 Universidad Autónoma de Madrid, Madrid E-28049, Spain; ‡ Institut de Ciència de Materials de Barcelona ICMAB-CSIC, Campus de la UAB, Barcelona E-08193, Spain; § 54449 Biophysics and Intelligent Matter Lab, Barcelona E-08193, Spain; ∥ Condensed Matter Physics Center (IFIMAC), Universidad Autónoma de Madrid, Madrid E-28049, Spain

## Abstract

Numerous respiratory viruses are transmitted via airborne microdroplets that frequently adhere to fomites. Understanding the behavior of these phenomenologically rich bio-material interfaces remains an open issue. Here, we tackle the complex interplay between glycans and protein conformational dynamics during adsorption onto polarizable surfaces, focusing on the potential of glycans as molecular interaction modulators. We employ molecular dynamics simulations to dissect the interactions of the Receptor Binding Domain (RBD) glycoproteins from different SARS-CoV-2 variants of concern (VoC), in both open and closed conformations, with polarizable planar interfaces. Advanced analysis using 2D space reveals distinct adsorption mechanisms depending on the initial loci of the glycan within the protein wall. Hydrophobic surfaces facilitate stable adsorption for both RBD conformations. Conversely, hydrophilic surfaces exhibit reduced adsorption, particularly for the closed-RBD, where glycans predominantly form hydrogen bonds. Glycans significantly modulate closed-RBD adsorption, either enhancing it by permanent tethering or impeding it depending on the initial conformation and protein mutations (Omicron). Results for the individual RBDs are consistent with scaled-up simulations for the complete spike ectodomain glycoprotein. Our findings unveil novel glycan-mediated adsorption phenomena and provide fundamental insights into glycoprotein–surface interactions, paving the way for understanding glycan roles in glycoprotein–fomite adsorption, protein aggregation, and recognition at polarizable biological interfaces.

## Graphical abstract

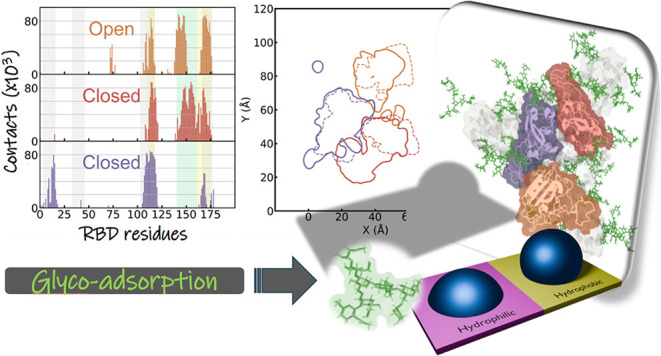

## Introduction

Understanding the interactions between viral proteins and their glycosylated sites has recently driven significant efforts by the molecular simulation community in comprehending novel intrinsic functions of glycans.^1–5^ These interactions are highly dynamic and environment dependent, as they have an amphipathic nature that allows glycans to interact with polar and nonpolar protein residues or surfaces.^4,6,7^ Particularly poorly understood is the question of how glycans modulate the adsorption of glycoproteins onto polarizable surfaces.^8^ Dissecting the interactions of glycans in the environment provided by hydrophobic and hydrophilic surfaces is crucial to uncovering the polysaccharide chain ability to enhance or weaken the adsorption process. A critical challenge in studying adsorption processes is defining proper initial conformations of the most flexible molecules (glycans) in the glycoprotein–surface system.^8,9^ Similar questions have been addressed to model the glycoprotein interaction in bulk water^3,9–11^ with receptor proteins^12–14^ and related setups.^8,15^ Each of these studies revealed important aspects of glycan interactions, such as the shielding mechanism of glycans toward the immune system defenses, which remarked the critical role of glycans during the vaccine development period against COVID-19.^1^

These findings provide momentum for addressing novel questions in glycosylation from a computational biophysics perspective. Among them is the effect of combining a commonly semiflexible molecule (proteins)^16,17^ with highly flexible ones (glycans).^18^ The challenge here is how to evaluate the effects of flexibility on the interaction in the 3D bulk. Polymer adsorption theory and simulations^19–21^ provide us with tools to evaluate this phenomenology by tackling the 3D interactions onto planar surfaces. This analysis benefits from simplified representations that enable us to guide the structural analysis of the adsorption configurations of biopolymers (glycoproteins, nucleic acids, and lipidic assemblies). Model polarizable surfaces^22^ offer an effective way to address the effect of hydrophobicity/hydrophilicity. They have been used in the characterization of adsorption patterns for biopolymers onto planar surfaces and membranes^21,23^ to tackle the electrostatic interactions of the VoC spikes with planar and charged surfaces^24^ and in our recent study of the adsorption of SARS-CoV-2 Receptor Binding Domains (RBD) for the WT, Delta, and Omicron variants of concern (VoC) in an open conformation.^8^ Recent experimental results with high-speed AFM^25^ characterized the interaction between the viral trimeric spike protein SpEct and inanimate surfaces, which revealed the highly dynamic transitions between open and closed conformations close to a surface. The highly dynamic interaction of the RBDs onto mica surfaces was previously observed by Hinterdorfer and coworkers for different VoCs.^11^

These experimental findings motivate the present study, where we employ molecular dynamics (MD) simulations to examine the interaction of individual SARS-CoV-2 RBD glycoproteins in the closed conformation and the spike ectodomain glycoprotein with model polarizable surfaces (Figure 1). Previously, we gained insights from MD simulations of the RBD open conformation adsorbing to the same surfaces.^8^ This analysis^8^ revealed stable adsorption to hydrophobic surfaces for the open-RBD glycoproteins, while hydrophilic surfaces were less prone to adsorption. One crucial property we identified was the key role of molecular flexibility at the interface, with the flexible RBM in the RBD-open configuration notably enhancing its adsorption strength.^8,26^ Motivated by this insight and considering the high flexibility of glycans, our main objective is to identify the glycan capabilities to enhance or block the glycoprotein adsorption strength for different initial closed-RBD conformations. While glycans do not significantly influence the adsorption of open-RBDs due to their location far from the protein–surface contact area,^8,27^ they happen to be crucial for tuning the adsorption in the closed-RBD conformations.

**Figure 1:**
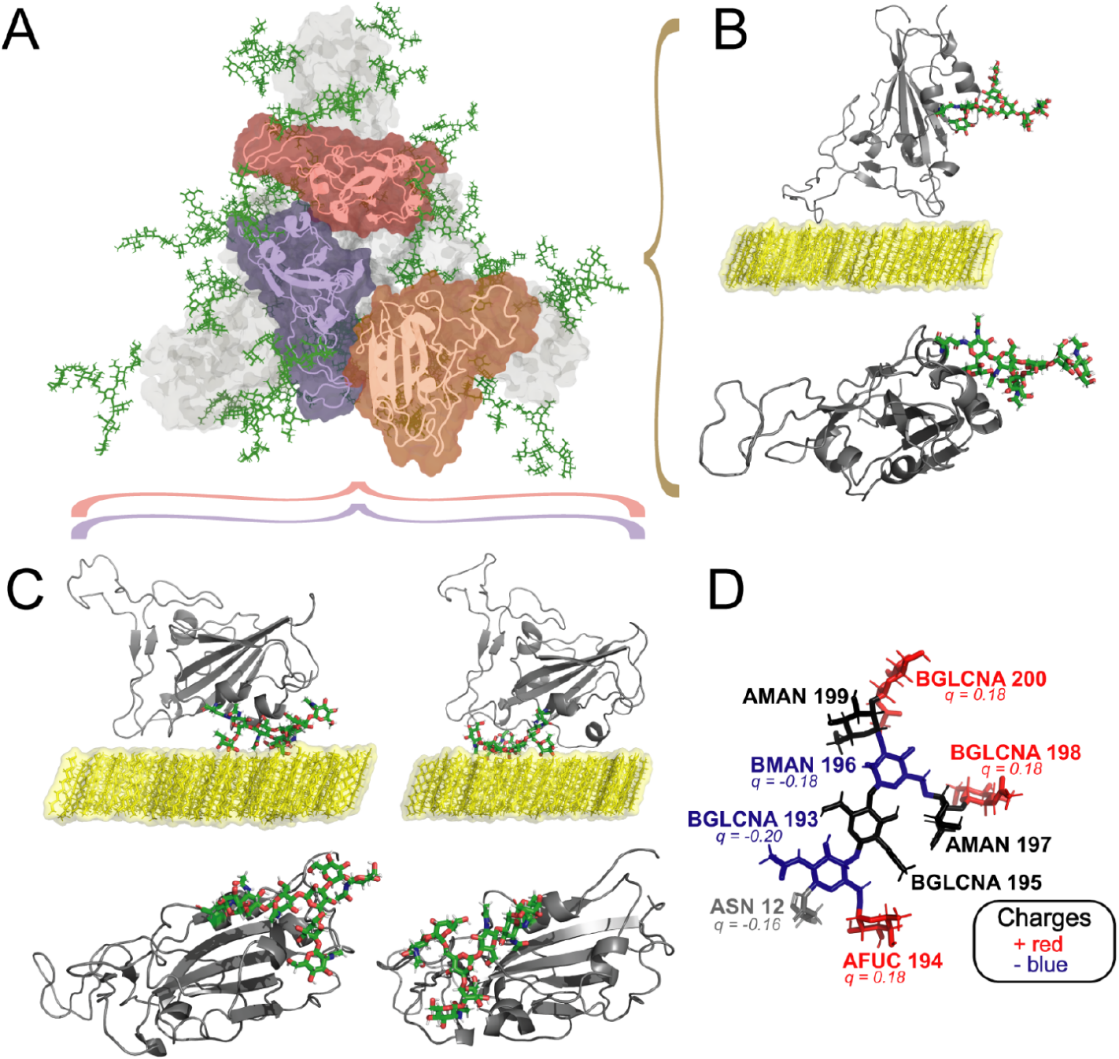
Protein–glycan complexes simulated in this study in the presence of polarizable bilayers (PBLs), which consist of a set of OH-group-tuned decanol molecules designed to reproduce different hydrophobicities. (A) Bottom view of the spike ectodomain glycoprotein (SpEct), with different colors indicating the three RBD monomers: one RBD is in its open configuration (orange), while the other two are in closed configurations (red and purple). The non-RBD regions of the SpEct are shown in gray, and glycans are depicted in green. (B) Front and bottom views of the isolated open-receptor binding domain (RBD) in the presence of PBLs, which were previously analyzed in detail in our earlier work.^8^ (C) Side and bottom view representations of the RBD in its closed configuration, showing the glycan positioned at the RBD head (left) and the RBM (right), respectively. Glycan colors in (B,C) follow an atom-type format, where green corresponds to carbons, red corresponds to oxygen, and white corresponds to hydrogen atoms. (D) A detailed view of the RBD’s glycan, including residue names of monosaccharides, is shown, with residues colored according to their partial charges based on the CHARMM36 force field; blue, red, and black colors represent negatively charged, positively charged, and neutrally charged glycan residues, respectively. The amino acid linking the glycan to the RBD is shown in gray.

Our approach builds up on the analysis of adsorption for single-RBD conformations to shed light on the adsorption process for the spike ectodomain glycoprotein (in open, closed, and closed OCC conformations). This strategy allows us to get a comprehensive understanding of the spike RBD glycoprotein adsorption first and, more importantly, to explore the interplay of the protein–glycan and glycan–glycan interactions together with the direct protein– and glycan–surface interactions. To this end, we employ existing^23,28^ and in-house-developed novel 2D analysis tools to quantify the morphological changes and the number, nature, and spatial distribution of the contacts formed as a function of the initial location of the glycan (in the RBD head or the Receptor Binding Motif—RBM—for the closed-RBD conformation, see Figure 1C) for each polarizable surface and RBD VoCs. The strategy of using planar interfaces to reduce the degrees of freedom of the short-range interactions in glycoproteins allows us to show the glycans’ high flexibility; to track in detail their interaction with the protein, other glycans, and the surface; and to reveal how these effects combine to drive or impede the adsorption.

The novel adsorption mechanisms identified in this work for glycoproteins and surfaces, together with the very recent development of methods that allow tracking glycans with angstrom resolution,^29^ provide the starting point to understand these processes in heterogeneous interfaces where glycans could induce glycoprotein–fomite adsorption, protein aggregation, multiprotein assemblies, and misrecognition of protein-based drugs.

## Results

### Morphological Changes and Contact Formation during Adsorption

The RBD glycoproteins simulated in this work consider three VoCs and three main conformations of the RBD, namely, open, closed-head, and closed-RBM (see Figure 1). In Figure 1D, we also display a close-up view of the N343 glycan^5^ residues and the polarities of its partial charges.

We produce trajectories for each system (see Methods for details) and use them to extract representative morphological changes and detailed information about the contacts formed during the adsorption process for both hydrophilic and hydrophobic surfaces.

The morphological analysis is based on the parallel and perpendicular radii of gyration, $R_{g∥}$ , $R_{g⊥}$ , calculated for trajectories based on 3 replicas and shown as individual dots, together with their time averages, in Figure 2. Figure 2A,C and B,D correspond to the two different initial positions of the glycan in the RBD glycoprotein in closed conformation, namely, the glycan is located at the head position (A,C) or at the RBM site (B,D), as previously described in Figure 1C. In terms of the ratio $\left\langle R_{g⊥}^{2}\right\rangle /\left\langle R_{g∥}^{2}\right\rangle$ (deformation) for the proteinaceous component of the glycoproteins, the closed-RBD conformations (white symbols) are clearly less flexible than the open ones (gray symbols): the relative percentage of the proportion of deformation ratios between closed-head and open is in the range ≈15–25% (depending on the VoC), while for closed-RBM and open it is ≈35–45% (see SI Table S3). These results align with percentual variations in the coiled structures ≈17–19% (variant dependent) of the secondary structures between the open and closed contact regions (see SI Table S10).

**Figure 2:**
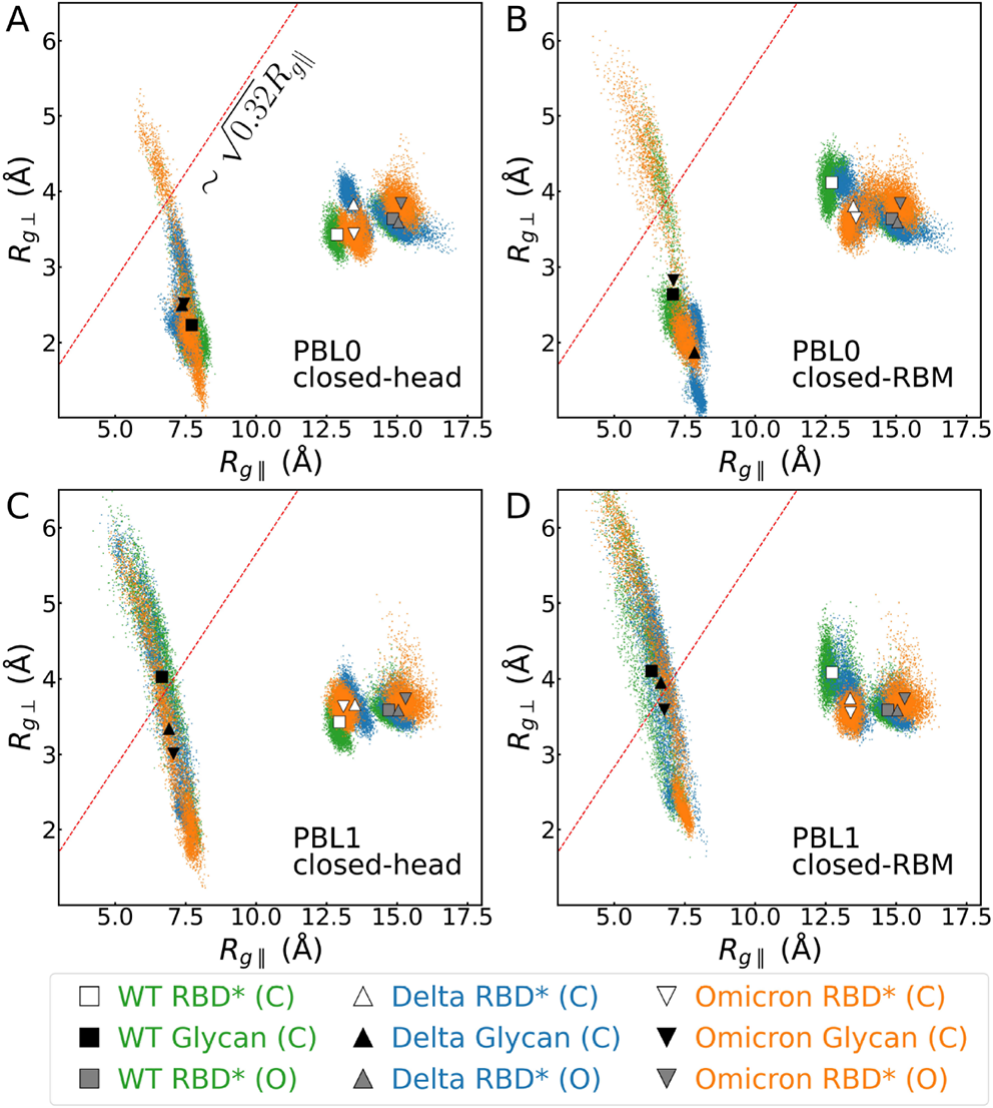
Parallel and perpendicular radii of gyration of the RBD protein in its open configuration in the absence of glycans (gray symbols), in its closed configurations (white symbols), and the glycan alone (black symbols) in the simulation with closed-RBD configurations. Small green, blue, and orange dots correspond to the data at each frame, and the symbols represent the mean values for WT (square), Delta (triangle), and Omicron (inverted triangle) variants, respectively. Red lines indicate the limit ∼ $\sqrt{0.32}R_{g∥}$ for the semiflexible polymer regime. Panels show closed-RBD with the glycan between (A) a hydrophobic surface and the RBD’s head, (B) a hydrophobic surface and the RBD’s RBM, (C) a hydrophilic surface and the RBD’s head, and (D) a hydrophilic surface and the RBD’s RBM. Note that the results for the radii of gyration of the proteins do not consider the whole protein but only the contact regions with the surface (cases marked with * in the legend). The exact values shown in this plot are also provided in SI Table S4.

In our recent work^8^, we reported that for the RBD in open conformation, the adsorption is stronger on the hydrophobic surface than on the hydrophilic surface. Note that in this work, we are considering the glycan–surface interactions (black symbols and corresponding distribution). $R_{g∥}$ and $R_{g⊥}$ along the trajectory (see Figure 2) show much higher fluctuations for the glycan compared to the protein, evidencing the highly flexible nature of glycans. However, the average values (black symbols for glycan, white and gray for the protein in Figure 2A and B) remain in the semiflexible regime. This result can be understood from the fact that in the great majority of these trajectories, the glycan is located between the protein and the surface. There are very subtle differences in the ratio $\left\langle R_{g⊥}^{2}\right\rangle /\left\langle R_{g∥}^{2}\right\rangle$ between the two glycan locations, namely, a slightly more flattened protein when the glycan is at the head (Figure 2A), resulting from the fact that RBM region is more flexible and spreads more than the head one (see also SI Figure S1). On the contrary, in the closed-RBM conformation (Figure 2B) the glycan prevents the direct interaction between the RBM region and the surface, and hence, its deformation during adsorption is around 1.5 times lower for both WT and Omicron (see SI Table S3).

For the hydrophilic surface, we observe a significantly different behavior in glycan adsorption for the closed conformations: the three VoCs present a huge fluctuation pattern, with the WT variant lying outside the boundary of semiflexible adsorption, while the Delta and Omicron variants are very close (Figure 2C and D). The glycan fluctuations can be associated with tethering conformations where the glycan interacts with the surface by forming hydrogen bonds, which are highly dynamic. Focusing now on the protein adsorption, there are clear differences between the two glycan locations for the WT: the ratio $\left\langle R_{g⊥}^{2}\right\rangle /\left\langle R_{g∥}^{2}\right\rangle$ increases 1.48 times (see SI Table S3) when comparing the head and RBM locations, reflecting the fact that the head-contact region is more hydrophobic than the RBM one. These differences are less pronounced in the Omicron variant, mainly due to the mutations bringing charged residues and intermittent formation of H-bonds.

Figure 3 compares both the total number of contacts and the contact area (see Methods) of the RBDs in 3 initial conformations: open, closed-head, and closed-RBM, interacting with two model surfaces. Divergent from the open RBD conformation,^8,27^ the N343 glycan in the closed conformations plays a crucial role in promoting or blocking protein adsorption.

**Figure 3:**
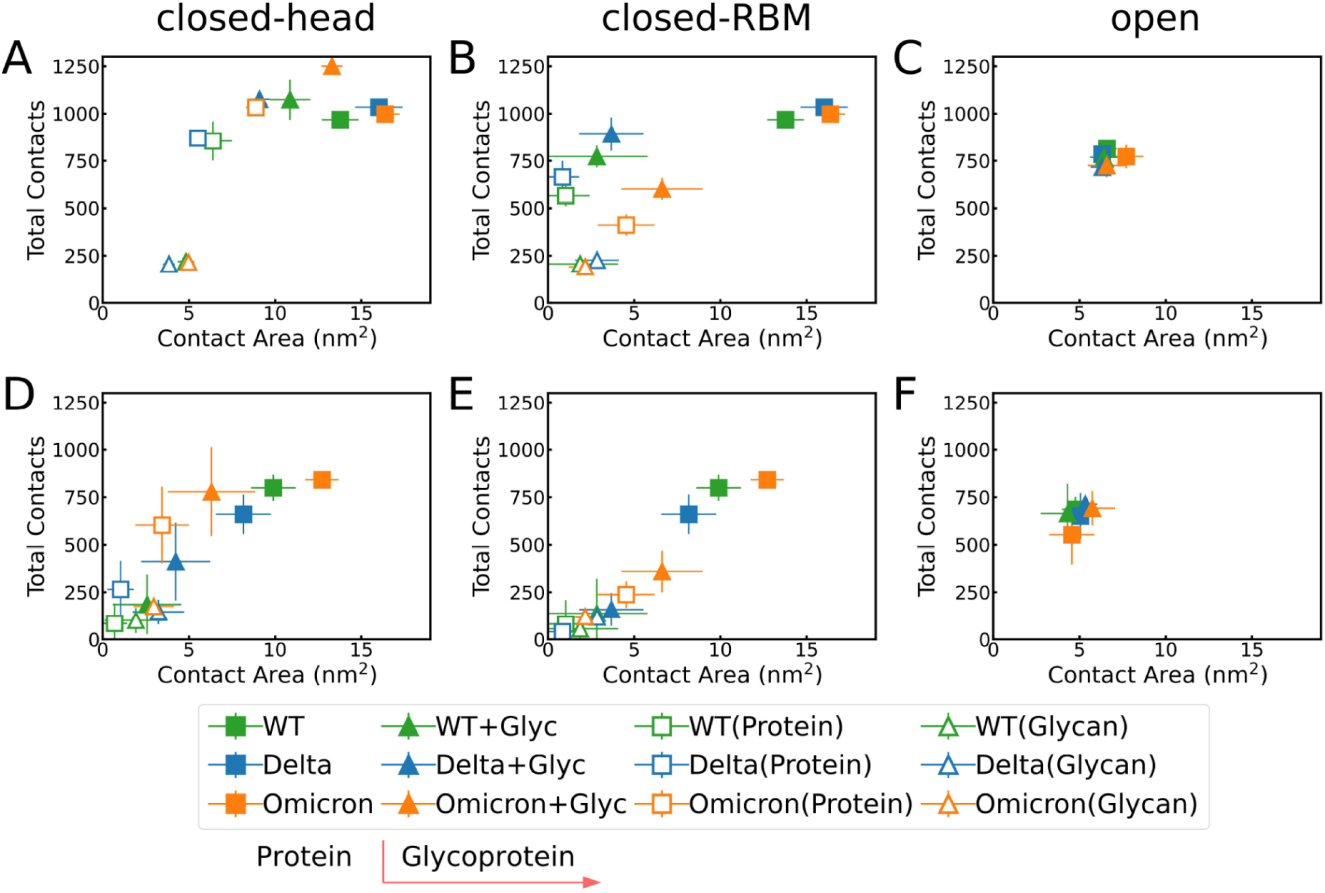
Total number of contacts versus contact area for interactions with (A-C) hydrophobic surfaces and (D-F) hydrophilic surfaces. Solid squares represent data from simulations without glycans, while solid triangles correspond to simulations including glycans. Empty squares and empty triangles indicate the contributions of the protein and glycan, respectively, in the protein–glycan complex simulations. Panels show results for the RBD in (A, D) the open configuration and in the closed configuration with the glycan positioned at the (B, E) surface–head RBD interface and (C, F) surface–RBM RBD interface.

Focusing on the two closed conformations for the hydrophobic case, the closed-head conformation doubles the contact area of the closed-RBM conformation (full triangle symbols denote the glycoprotein in Figure 3). However, the total number of contacts decreases from ∼20% (WT and Delta) to ≈50% (Omicron) when the glycan is initially located at the RBM region. As expected, this difference is related to the mutations in Omicron’s RBM region, which make it more hydrophilic (see SI Tables S1 and S2). When the glycan flattens between the protein and the surface, it forms hydrophilic interactions with the protein, preventing protein adsorption on the surface. This conclusion is confirmed by the separate analysis of glycan and protein (empty symbols in Figure 3) that shows for closed-RBM a significant reduction in the protein contacts and in its contact area. These changes are drastic for WT and Delta (reducing them to minimal values), while in the case of Omicron, the protein contact area is only half that of the closed-head case.

Both the total number of contacts and the contact areas reduce for all VoCs at the hydrophilic surface (compared to the hydrophobic one), as shown in Figure 3D,E and F. Comparing the two glycan locations, both quantities decrease when moving from the closed-head to closed-RBM conformations, but the reduction in contact area is not drastic due to the fact that the hydrophilic interactions (associated with the formation of hydrogen bonds) are dominated by the glycan, as discussed in the Fluctuation Analysis and Hydrogen Bonding.

The footprints or 2D densities of the top 5 residues—the location of their center-of-mass (COM) along the trajectory—for WT and Omicron forming more contacts with the hydrophobic surface, together with the position of the glycan’s COM, are shown in Figure 4. The data extracted from the trajectories for each system (closed-head, closed-RBM, and open, as specified in SI Table S12) reveal a significant dependence on the conformation and the initial glycan location. The insets in Figure 4 display the time-dependent distances with the surface of these residues (blue) and the glycan (pastel-red curve).

**Figure 4:**
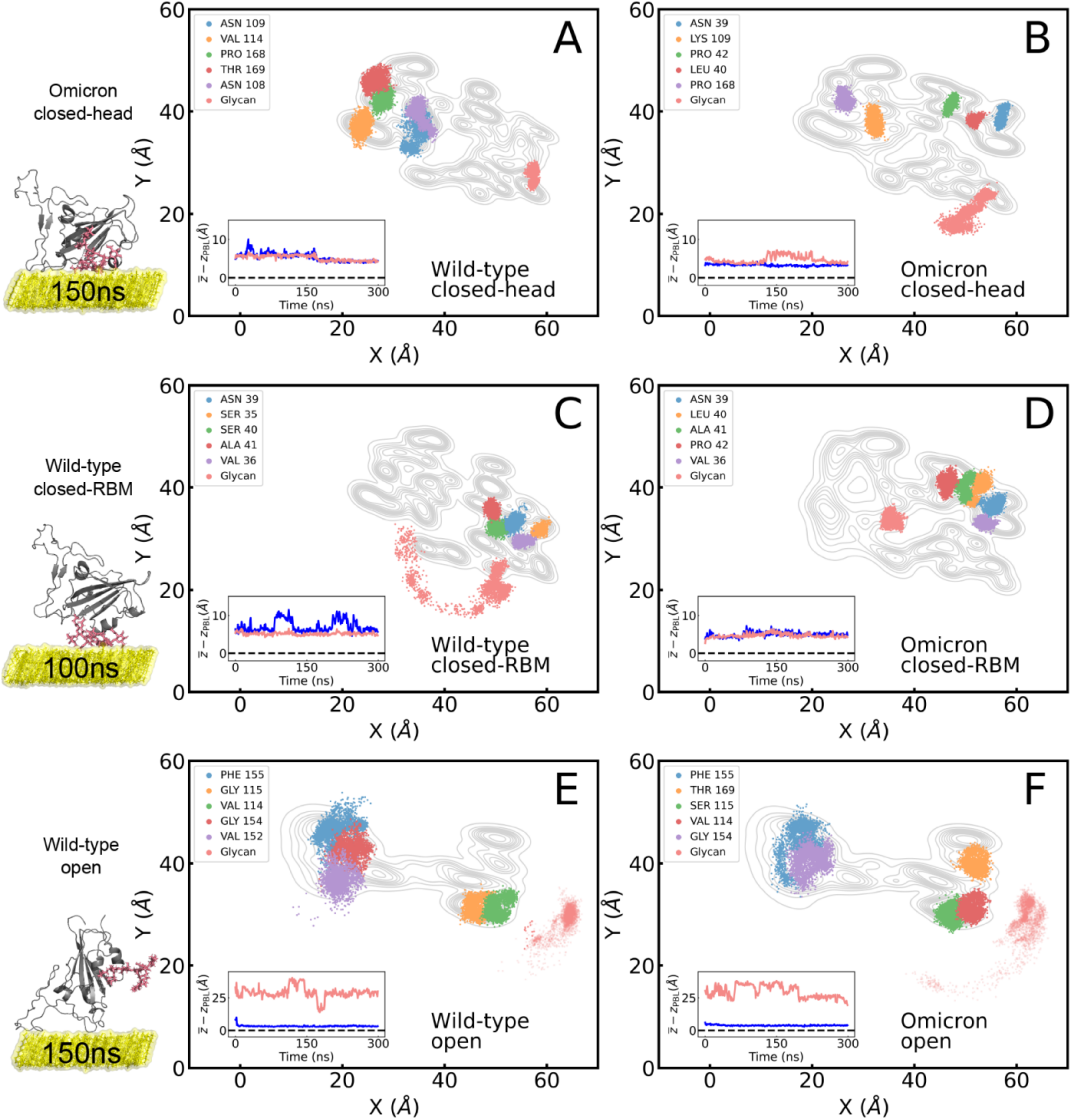
Footprint of the top five protein residues with the highest number of contacts to the hydrophobic surface and the center-of-mass (COM) of the glycan for the closed- and open-RBD configurations for WT and Omicron variants. Insets show the average distances of these residues (blue) and the glycan (red) from the surface. Panels display results for (A, B) closed-RBD with the glycan between the RBD head and the surface, (C, D) closed-RBD with the glycan between the RBM and the surface, and (E, F) open-RBD configuration. Contours of the contact regions of the closed and open RBDs are shown as reference maps for the protein. On the left of the panels, representative snapshots of the indicated simulations are shown.

In the closed conformations, both distances mostly align with each other (see the insets in Figure 4A–D). This behavior is completely different from that in the open conformations (Figure 4E and F), tackled in our previous work^8^ where the glycan is attached on the side (Figure 1B). The size of the RBD’s sidewall is commensurate with the length of the modeled polysaccharide chain, precluding a direct interaction between the glycan and the surface for open conformations. The situation is different for the closed conformations, where the glycan is initially located between the protein and the surface. Here, depending on the glycan’s initial loci and VoC, we observe a diverse phenomenology. For the close-head configuration (Figure 4A), the 5 top interacting residues are located at the RBM for WT, while for Omicron, 3 of them are found at the head side. This distinct behavior results from the combined influence of mutations at the head site, which became more hydrophobic (SI Table S1), and the glycan’s mobility. The glycan–surface distance (pastel-red curve inset in Figure 4B) illustrates the process where the glycan squeezes out from its initial location toward the liquid–surface interface and jumps out-of-contact for over one-third of the trajectory.

A quite different behavior is found in the closed-RBM case (Figure 4C and D). Here, the glycan flexibility contributes to holding the WT glycoprotein in contact with the surface during different unbinding events (Figure 4C). The protagonists of those events were 2 Serines (ResIDs 35 and 40) located at the WT’s head. In Omicron, two mutations (LEU 40 and PRO 42) enter the top 5 contacts and contribute, with their nonpolar character, to provide a stable adsorption during the whole trajectory (see inset of Figure 4D). Representative snapshots of the adsorption behavior for the hydrophobic surface are embedded in three cases, namely, glycan squeezing-out (Figure 4B), tethered adsorption (Figure 4C) and no adsorption or glycan interacting with water (Figure 4C). Further illustrations of each of the trajectory snapshots for this figure are in SI Figure S4.

The results for the hydrophilic surface are shown in SI Figure S2. Here, the adsorption behavior follows, in general, an oscillatory pattern similar to the one previously reported for the open conformation.^8^ However, in the closed conformations, the glycan plays an active role, trying to keep the glycoprotein in contact with the surface (see the snapshots in SI Figure S5). As can be expected from the almost identical sequences in the contact region (see SI Tables S1 and S2), the adsorption behavior of Delta (SI Figure S3 and snapshots in SI Figure S5) is very similar to WT on both surfaces. To summarize this analysis, we quantify the angular interactions of each monosaccharide of the N343 glycan (SI Figures S6 and S7) in order to further confirm the main message: the amphipathic behavior of this glycan on either hydrophobic surfaces (with angles less than 45°) or hydrophilic ones (with larger angles).

### Fluctuation Analysis and Hydrogen Bonding

The identification of the residues with the highest RMSF values (Figure 5) provides further insight into the contact analysis presented above. Correlating the number of contacts with regions of larger fluctuations illustrates whether fluctuations promote more contacts at the interface or if those contacts are rather based on highly localized short-range interactions with less flexible protein regions. Figure 5 presents, for WT and Omicron adsorbed on the hydrophobic surface, the footprints of the five protein residues with the highest RMSF values, the areas covered by them and by the glycan COMs (see legends for quantitative values), and a profile with the RMSF values for all protein residues in the contact region. The same analysis is available in the supporting information for the hydrophilic surface (Figure  S8) and the Delta variant (Figure  S9).

**Figure 5:**
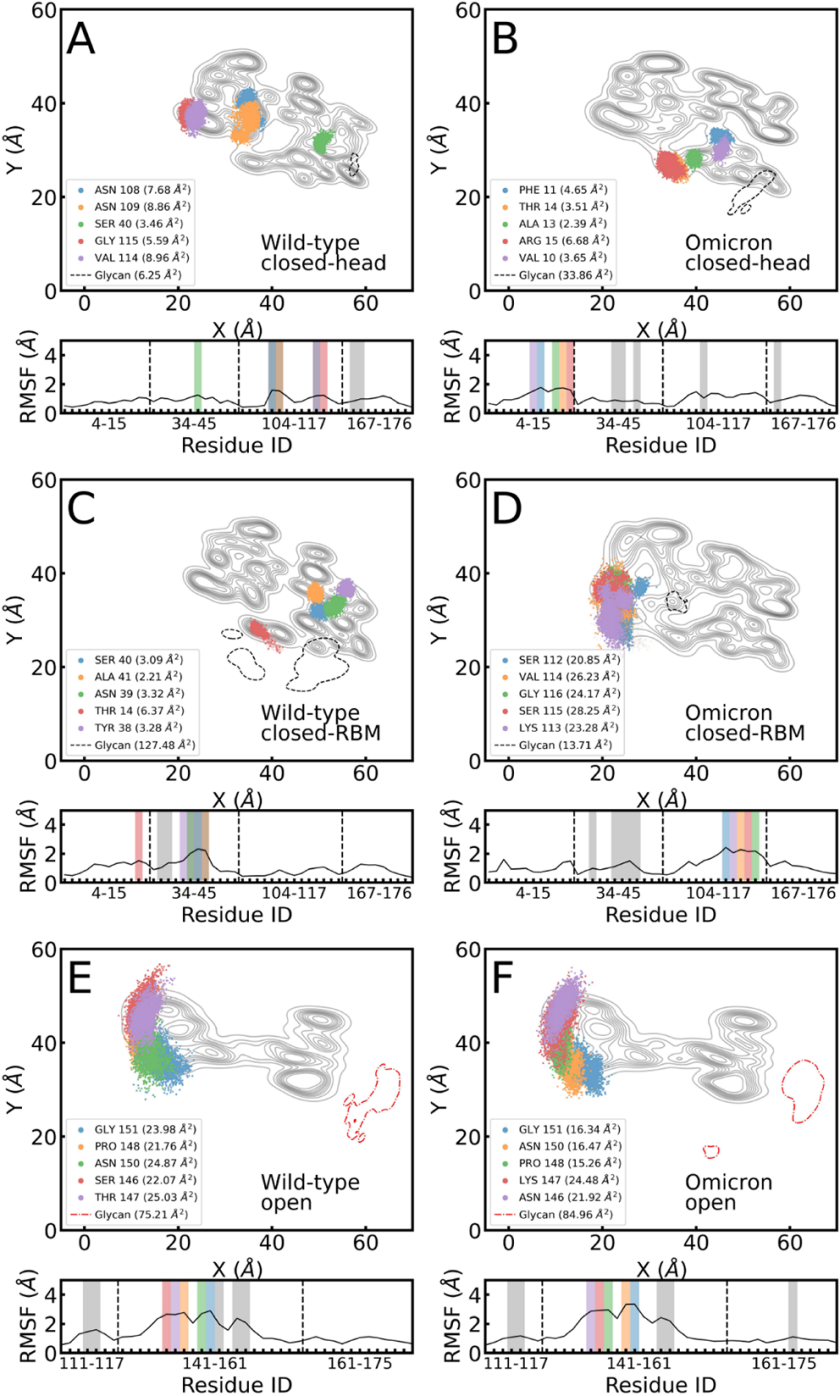
Footprint of the top five protein residues in the contact region with the highest RMSF values and the area covered by glycan (dashed line) for the closed-RBD and open configurations onto the hydrophobic surface for WT and Omicron variants. Panel ordering is consistent with Figure 4, e.g., closed-RBD with glycan (A, B) in head, (C, D) in RBM, and (E, F) open-RBD. RMSF values for residues in the contact region are shown below each footprint, with colored regions highlighting the residues shown in the footprint, and gray regions corresponding to residues with the highest number of surface contacts (Figure 4). Red dot-dashed lines in (E) and (F) are a reminder that the glycan in the open conformations is not adsorbed (normal distance >14 Å).

According to the 2D fluctuation areas for the closed-head conformation (Figure 5A and B), the glycan in WT fluctuates less at the interface than in the Omicron. The RMSF profile for the closed-head conformation in Omicron shows that all of the residues with the highest RMSF are located in close proximity to the area covered by the glycan. None of these residues are among the top 5 in the number of contacts (Figure 4B). This result highlights how glycans can sterically prevent the approach of residues to the surface, particularly for the 3 hydrophobic residues (VAL 10, PHE 11, and ALA 13) captured in Figure 5B. This behavior is not found in WT (Figure 4A), where 3 (ASN 108, ASN 109, and VAL 114) out of 5 residues with larger fluctuations at the interface are also the ones achieving more contacts.

For the WT closed-RBM conformation (Figure 5C), glycan fluctuations are in the spotlight as their area is 2 orders of magnitude larger than those of the protein residues with the highest RMSFs. Here, glycan fluctuations are effectively promoting the adsorption of residues because the glycan extends its interaction with the surface through different areas than the location of residues at the RBM. As seen before for the closed-head conformation: again, 3 (ASN 39, SER 40, and ALA 41) out of the 5 most fluctuating residues coincide with the residues with the most contacts (Figure 4C). Here, the adsorption is mainly driven by the head region that includes the most hydrophobic residues, which also pull neighboring hydrophilic ones during their adsorption process. In the case of Omicron variant, Figure 5D illustrates the effect of neighboring residues (ResIDs: 112–116) in promoting fluctuations: Larger RMSFs are found in the vicinity of mostly hydrophilic residues at the RBM region, including the GLY-to-SER mutation of residue 115. On the contrary, interactions with the hydrophobic surface (mutations turned the head region in Omicron more hydrophobic) led to significantly smaller RMSF values, around half of the top ones (gray areas in the RMSF profiles).

The open conformations (Figure 5E and F) are, by far, the ones where protein residues fluctuate the most, with RMSF values much higher (maxima around 3 Å) than those for the closed conformations (which remain close to 2 Å), with the exception of the closed-RBM Omicron’s case. These results illustrate the case where the glycan, due to its location on the side of the RBD protein, plays a minor role in the RMSF footprints and is shown for comparison.

Nonetheless, comparing their RMSF profiles, the open conformations for both variants reach values around 3 Å, while the closed conformations remain in the vicinity of 2 Å. An important remark for the glycan RMSF areas shown for the open conformation is that it is only a projection on the surface place. However, the *z* distance is not within 14 Å from the surface and hence is shown in red dot-dashed lines (see Figure 5E and F).

Figure 6 shows the protein and glycan residues that preferentially form hydrogen bonds (H-bonds) with the hydrophilic surface for the WT and the Omicron in the three conformations described above. Circles are centered at the COMs of the residues, and their size represents the percentage of the frames along the trajectory where this residue established an H-bond. Contour lines for the corresponding RBD protein and the glycan (dashed line) provide a reference to identify the areas that dominate H-bond formation. At variance with the open conformations studied previously^8^ and represented here by a single trajectory (Figure 6E and F), H-bond formation for the closed configurations is dominated by the N343 glycan (see Figure 6 and the SI Tables S5 and S6 for quantitative values).

**Figure 6:**
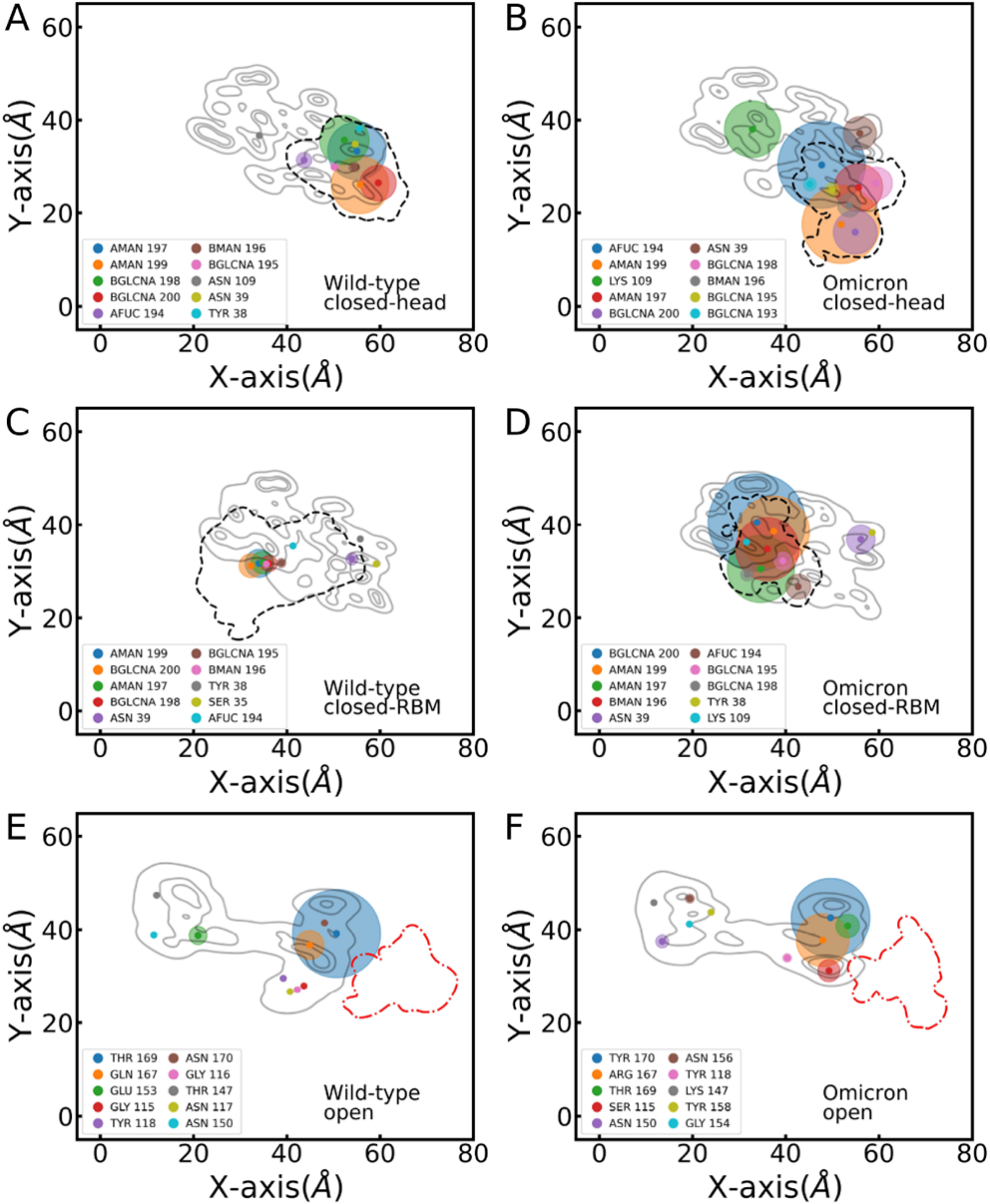
Hydrogen bond occurrence between the protein and the surface during the simulation time (shown as %). H-bonds are represented in circles centered at the center of mass of each residue, with proportional size to the percentage of H-bonding during the trajectory. The three contour levels in gray represent the mean positions of the protein, and the single contour in segment-black indicates the mean position of the glycan. Panel ordering is consistent with Figures 4 and 5, with WT in the left panels and Omicron in the right panels, i.e., closed-RBD with glycan (A, B) in head, (C, D) in RBM, and (E, F) open-RBD.

Starting with head-RBD, seven glycan residues are the ones with the highest percentages in WT (Figure 6A). Mutations from hydrophobic to hydrophilic residues at the RBM in the Omicron compete with the glycan, with the LYS 109 residue appearing in the third position and reaching nearly 35% of H-bonds along the trajectory (Figure 6B). This result explains the significantly larger contact area of the Omicron variant over the WT discussed above (see Figure 3). Protein mutations clearly also affect the glycan conformation and H-bond formation, with the glycan AFUC residue reaching, in the Omicron case, a percentage around 5 times that of WT. The glycan dominance is also reflected in the H-bond distribution within the RBM region: the balance found between the two groups (left and right sides) in the open conformation (see Figures 6E and 9 in ref 8) is lost in the closed conformations, where the glycan clearly defines the H-bonding-rich interaction regions. We observe similar trends for the closed-RBM conformation, albeit with a significant reduction in H-bond formation in the WT compared to the closed-head. In fact, the closed-RBM WT H-bond occurrence clearly shows the versatility of the glycan to monopolize most H-bond interactions. The study of the Delta variant (SI Figure  S10 and Table S7) leads to the same conclusions, with a clear predominance of the glycan in H-bond formation for the closed conformations and a rather balanced H-bond occurrence for the open RBD.

### Glycan Interactions with the Surfaces and the Protein Walls

After analyzing the adsorption of the glycoprotein as a whole, we focus our attention on the glycan and characterize, at the level of a single monosaccharide, the interaction with the surface and protein during adsorption. Figure 7 shows, in the closed conformations of WT and Omicron, the specific contacts formed by each of the glycan residues with the hydrophobic (Figure 7A and B) and hydrophilic (Figure 7C and D) surfaces. General trends, like the drastic reduction in the number of contacts in the hydrophilic case irrespective of the VoC, have already been identified in the literature for proteins^8^ and surfactants.^22^ In this work, the analysis provides similar results for the glycoprotein in closed conformation, showing a reduction in the number of contacts. Figure 7 confirms that these trends also apply to N343 glycan, demonstrating its amphipathic character. Here, the in-depth analysis at the residue level provides more insight into the role of the glycan in the process. While for the hydrophobic surfaces, there are no significant variations in the number of contacts associated with each of the monosaccharides of the glycan chain, they can be clearly spotted in the hydrophilic case, particularly for the WT variant. This indicates that the glycan is not completely adsorbed, and there are intermittent interactions with the surface where the last monosaccharides of the chain (the ones located further from the anchoring point to the protein residue ASN 12) help tether the WT-RBD (SI Figure S5A). This is also consistent with the high fluctuations of the parallel and perpendicular radii of gyration of the glycan (Figure 2). For the Omicron in closed-head conformation (Figure 7C), the enhanced hydrophilic character of the RBM adds to the glycan contact formation with the hydrophilic surface and hence promotes adsorption (SI Figures S5B). For the closed-RBM conformation, the head region—with its more hydrophobic character with respect to WT—is exposed, which hinders Omicron adsorption on the hydrophilic surface with respect to the closed-head conformation. The analysis for the Delta variant (SI Figure S11) shows again the amphipathic behavior of the glycan, which is further confirmed by our analysis of the angles of the sugar rings with respect to the surface normal (SI Figure S7).

**Figure 7:**
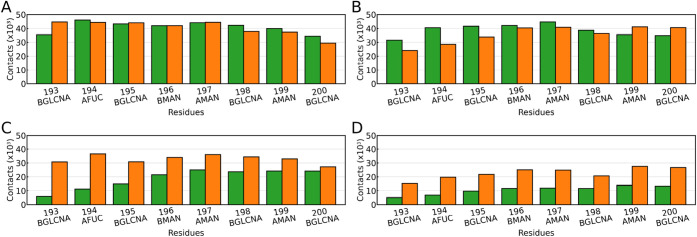
Glycan contacts with the surface when positioned between the surface and (A, C) the RBD head or (B, D) the RBM region. Panels (A) and (B) correspond to a hydrophobic surface, while (C) and (D) correspond to the presence of a hydrophilic surface. Results for the wild-type (WT) variant are shown in green, and for Omicron in orange.

Now, we focus on the glycan–protein interaction. Contact maps for each of the monosaccharides in the N343 glycan and the protein residues in the contact region for WT (top) and Omicron (bottom) plus the two model surfaces are shown in Figure 8. They display the ratio between the number of frames along the trajectory where the contact is present and the total trajectory frames. All contact maps include a green dashed line separating the Head and RBM regions. The general difference between closed-head and closed-RBM conformations is that the glycan at the head region interacts mostly with the protein residues in this region (Figure 8A, C, E, and G). This can be partly understood by the fact that the glycan attaches to residue ASN 12, located in the head region.

**Figure 8:**
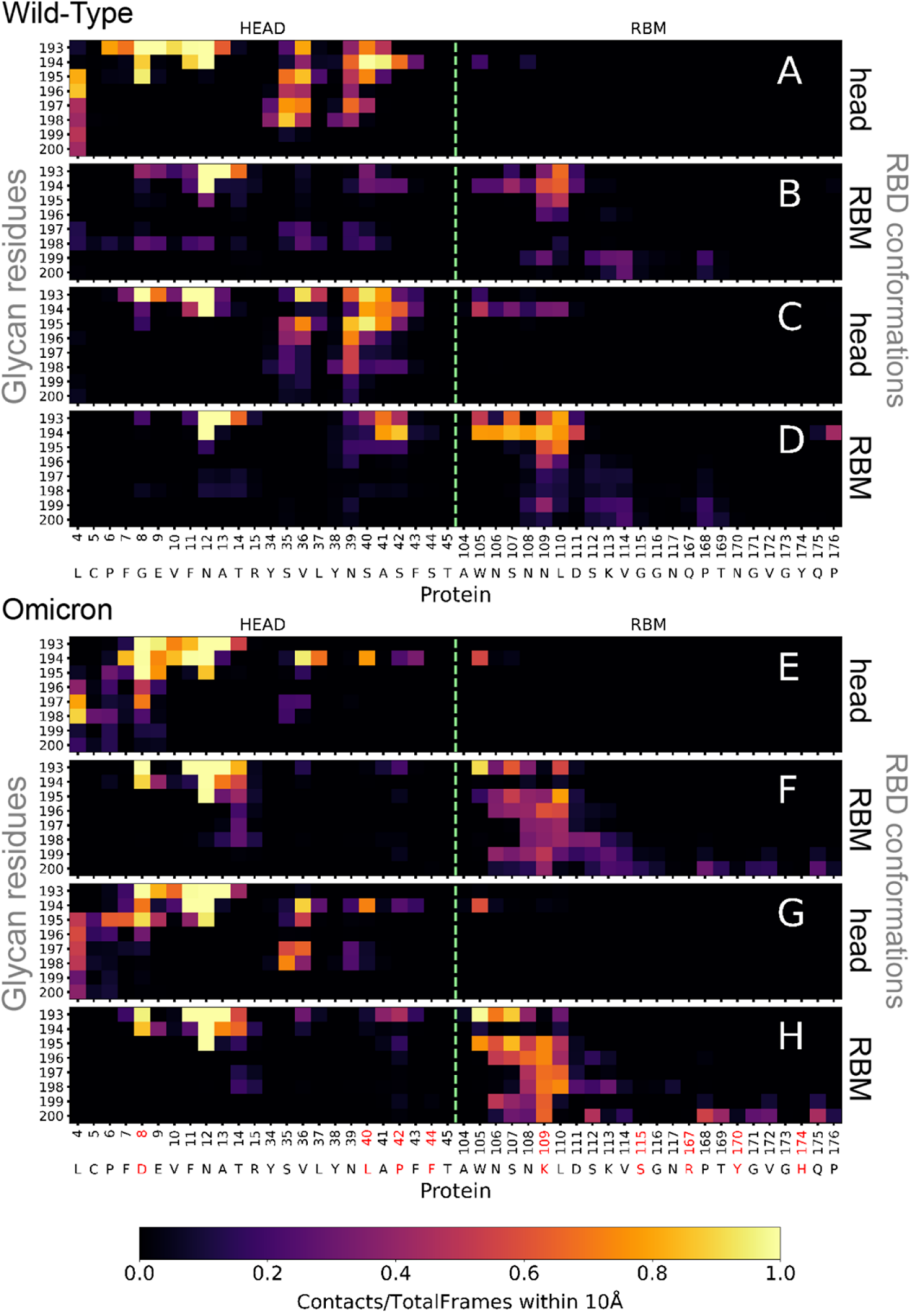
Contact maps between glycan and protein residues within the contact region for (A-D) WT and (E-H) Omicron. Panels (A-F) show results from simulations with a hydrophobic surface, while panels (C-H) correspond to a hydrophilic surface. In panels A, C, E, and G, the glycan is positioned between the RBD head and the surface; in panels B, D, F, and H, it is located between the receptor-binding motif (RBM) and the surface. A horizontal segmented lime green line in each map indicates the boundary separating residues in the RBD head from those in the RBM region of the RBD. Contacts are considered as residue–residue distances under 10 Å.

Hence, even for the closed-RBM conformation, the glycan has to interact with parts of the head region, mainly the protein fragment between residues 7 and 15. This can be clearly seen for Omicron in Figure 8F and H. Such a tendency is not as strong for WT, which we attribute to the larger fluctuations of the glycan on the surface and toward the head region, as shown in pastel-red footprints (Figures 4C and S2C). The closed-RBM conformations also show key differences between WT and Omicron due to the mutations (marked in red in the *x*-axis) that make Omicron’s RBM more hydrophilic and lead to an increase in the total number of contacts and to extend them to all the protein residues in positions 104 to 115 (Figure 8F and H). WT, without those additional hydrophilic residues, has a much narrower distribution of contacts (Figure 8B and D). The latter panel D shows a very strong interaction with AFUC 194, which we presume is an effect of the protein oscillatory adsorption on the hydrophilic surface. Table 1 quantifies the number of contacts (distances below 10 Å), taking into account the electrostatic character of the residues (positively (CP) and negatively (CN) charged, nonpolar (NP), and uncharged polar (UP)). Values are normalized to the product of the total number of simulation frames and the total number of glycan residues. Numbers in parentheses correspond to the total number of protein residues in each of the four categories. In the case of the glycan, we have three positively charged monosaccharides, two negatively charged, and three neutral ones.

**Table 1:**
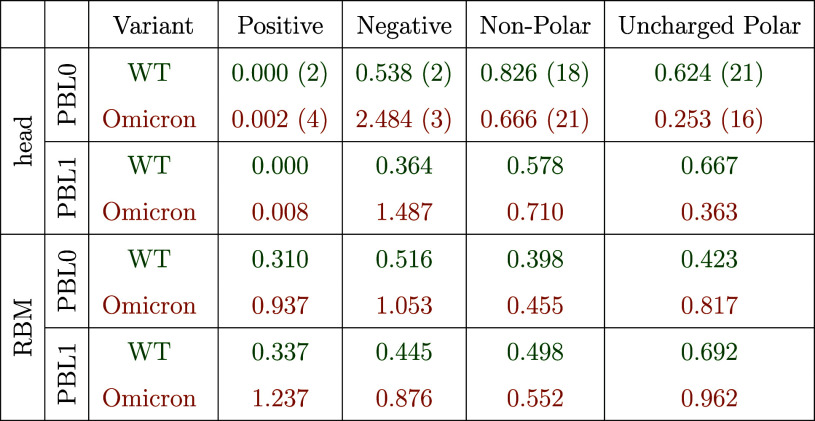
Contact Frequencies between Protein Residues in the Contact Region and the Glycan, Grouped by Residue Type (Positively Charged, Negatively Charged, Nonpolar, and Uncharged Polar) for WT and Omicron with a 10 Å Contact Cutoff^a^

a Values are normalized by the total number of glycan residues and the number of simulation frames considered. A value equal to the number of residue in a given type (in parentheses) corresponds to the theoretical case where there is at least one contact of glycan residue with one protein residue. Note that the glycan residue can have more than one contact with protein residues. Glycines have been excluded from the count due to their low polarity.

Starting at the closed-head conformation on the hydrophobic surface (PBL0), the WT glycan increases contacts with negative residues: the average number of contacts with the two negative residues is of the same order as those with 21 uncharged-polar and 18 nonpolar residues. For the Omicron, negative contacts completely dominate. This is clearly a collective effect that goes beyond the presence of one more negatively charged residue (ASP 8) and is probably due to the presence of two adjacent negatively charged residues (ASP 8 and GLU 9) located very close to the glycan attachment location (ASN 12). The same trends are found for the hydrophilic surface.

The closed-RBM conformations show a dramatic increase in the number of contacts with positively charged (CP) residues. Again, this effect is more pronounced in Omicron and goes beyond the change in the total number of CP residues (2 in WT, 4 in Omicron), as previously discussed in other works focused on the entire RBD electrostatic interactions.^24,30,31^ The neighboring locations of LYS109 and LYS113 probably contribute to this enhancement, which is more pronounced on the hydrophilic surface. Moreover, the mutation to negatively charged ASP 8 in Omicron (formerly neutral GLY 8 in WT) shows strong interactions with the positively charged AFUC 194, which is very close to the anchoring monosaccharide (see the fluctuating areas of the glycans in Figures 5D and S8D). The conformation of the glycan at the RBM position could also screen the contact with negative residues for Omicron. Contacts for neutral residues decrease for nonpolar residues and significantly increase with uncharged polar ones in Omicron. These results suggest that the local charge distribution of the glycan is key to understanding the dynamics and interactions of glycans, and not only the globally neutral character of this polysaccharide, as is the case for purely proteinaceous systems.^32^ Note that the analyses for the contact maps (Figure 8) and contact frequencies (Table 1) use a cutoff distance of 10 Å. We chose this cutoff for illustrative reasons of the protein–glycan interactions to understand the closest contact, as a consequence of both tackled glycan location at the RBD closed interface. In the Supporting Information, we show the analyses for a cutoff of 14 Å (SI Figure S12 and Table S8), which, given the nature of such a flexible and heterogeneous interface, makes its contact visualization difficult. Further analyses for the Delta variant are also included in SI Figure S13 and Table S9.

### Upscaling of the RBD to the Spike Ectodomain Glycoprotein on Polarizable Surfaces

In order to validate our minimal model centered on the spike protein RBD, we extended our study to the complete spike ectodomain protein. The spike trimer conformation we chose is the open, closed-head, or closed-RBM, as it represents over 55% of the open-to-closed RBD distribution.^33^ Going beyond the small system has twofold objectives: first, to verify that the initial conformations of the glycan at the RBD we assume (closed-head and closed-RBM) can be found in the company of their neighbors’ additional open and closed RBD conformations. In Figure 9, we analyze the 2D densities as contours of the bottom RBD trimer comprising the spike protein and quantify the number of contacts made by the spike ectodomain at the contact regions studied in our small RBD-centered system. For the hydrophobic surface shown in Figure 9A and B, we recognize that the contour areas of the small system (dashed lines) are mostly contained within the spike ectodomain system (solid lines). The number of contacts coincides with the targeted residues in our single-RBD studies by 80 ± 10%. The behavior of the glycoprotein on the hydrophilic surface (Figure 9C and D) also shows a coincidence of contour areas, but to a lesser extent than the hydrophobic surface. Similarly, the contacts mostly match the residues in the range of 100–175. Regarding the coincidence of glycan conformations, we quantify the number of contacts between the RBD glycans and the proteins from the spike ectodomain simulations in Table 2. Strikingly, the conformations we studied at the single-RBD level for the closed RBD account for more than half of the total interactions between the protein and glycans at the spike ectodomain level for WT and Omicron, and in both conformations, open and closed (see Table 2).

**Figure 9:**
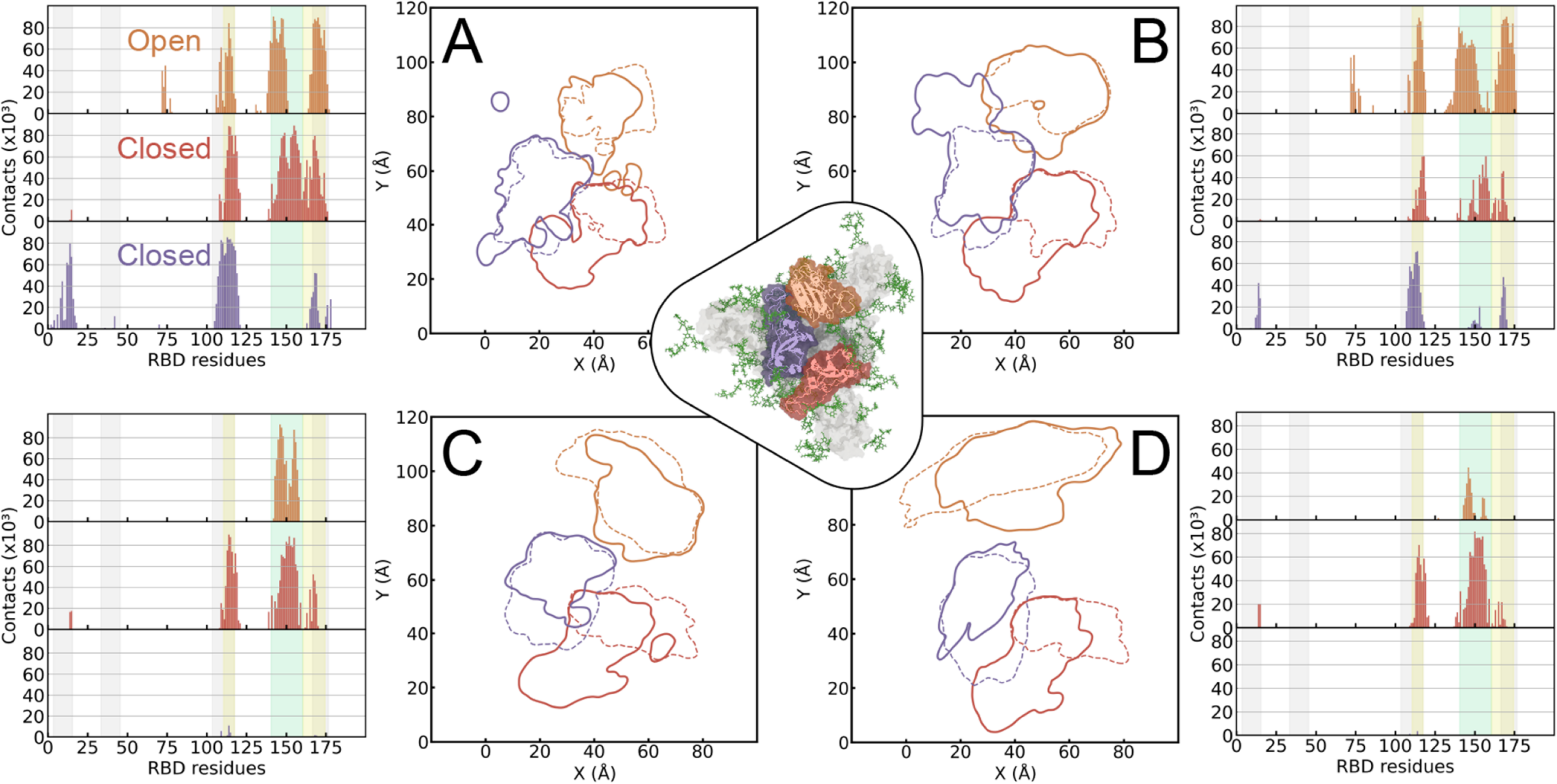
RBD adsorption in an open-closed-closed-spin ectodomain protein configuration. Each panel shows a contour plot representing the contact regions of the three spike RBDs in the presence of PBLs and contact histograms of the RBD residues. In the contour plots, dashed lines indicate the contact regions analyzed in the isolated RBD simulations, and solid lines enclose the top 50 residues with the most surface contacts. In the contact histograms, green and orange regions highlight the two contact regions previously considered for the open-RBD configuration.^8^ In contrast, gray color highlights the contact region of the closed configuration analyzed in this manuscript. RBD colors are consistent across the plots with the dark orange RBD corresponding to the open configuration. Panels (A) and (B) show the results for the WT variant in the presence of hydrophobic and hydrophilic surfaces, respectively. Panels (C) and (D) correspond to the Omicron variant under the same conditions. In the center of the figure is a representation of the SpEct glycoprotein.

**Table 2:** Glycan Interactions for Spike Ectodomain Glycoprotein-PBL simulations^a^

RBD type	Glycan w/ RBD’s head	Glycan w/ RBD’s RBM	Glycan w/ other glycans	Glycan w/ remaining SpEct
WT Open	66.31	14.97	0.46	18.25
Omicron Open	72.91	9.72	9.32	8.05
WT Closed	58.22	7.35	0.42	34.01
Omicron Closed	63.76	12.87	4.05	19.32

a This table reports the percentage contribution of contacts with the head and RBM region of the attached RBD, as well as with other glycans and the remaining protein residues of the spike ectodomain glycoprotein, with a distance cutoff of 14 Å. Note that the snapshots of each system are found in SI Figures  S14–S17.

The remaining interactions for the N343 glycan involve other regions of the glycoprotein complex (besides their corresponding RBD contact regions, either head or RBM), namely, the non-RBD spike ectodomain protein residues and the non-RBD other glycans (beyond the N343 one placed at the RBD site). Remarkably, those 2 interactions do not exceed 35% of contacts for the spike ectodomain OCC conformation. Another interesting result is that the closed-head conformation is at least 4 times more frequent than the closed-RBM conformation at the spike ectodomain level. This is partly attributed to the fact that the glycan is attached in the head region. However, it is also related to the availability of the contact regions, as we calculate that the proteinaceous contact frequencies with the surfaces for the spike ectodomain complex (see Figure 9) are mainly located at the RBM site. In other words, it flags this site as busy , while the head region contains mostly fewer contacts (Figure 9). Another interaction that has been made tractable within this analysis is the interaction of other glycans of the spike ectodomain (WT or Omicron; see SI Table S11) with either the head and/or RBM regions, which represent a maximum of ≈19% for WT (sum of the second column for the open conformation) and ≈33% (sum of the second column for the closed conformation) for Omicron. The external glycans also have a clear preference to interact with the head site of both variants and any conformation. Some snapshots illustrating the interactions of the “glycan context” are shown in SI Figures S14–S17.

## Discussion

We examined the role of glycans in the adsorption of RBD glycoproteins onto planar hydrophobic and hydrophilic surfaces. We used model surfaces in order to generalize our results to specific short-range interactions, which capture the effect of mutations on different RBD glycoprotein VoCs in their contact regions. Our investigation highlights the glycans’ dual capacity to act either as a glue that dominates the whole RBD glycoprotein adsorption strength via dynamic tethering patterns or as a blocking element, preventing the protein–surface interactions. The existence of several mutations in the RBD glycoproteins provides the perfect playground to spatially understand the effect of mutations in the contact regions via short-range interactions, particularly between the WT and the Omicron variants. Here, the challenge is to identify the location of those interactions, which we accomplish with the help of our planar surface model that enables the evaluation of those interactions with fewer degrees of freedom than in a bulk representation. For the “on-surface” analysis, we chose a combination of polymer adsorption theory^19–21^ with novel advanced 2D analysis^23^ on top of specifically developed analysis routines to evaluate the context of glycans and proteins during adsorption. These analyses allow us to identify and quantify the rich phenomenology of a highly flexible molecular complex upon adsorption. The deformation (ratio $\left\langle R_{g⊥}^{2}\right\rangle /\left\langle R_{g∥}^{2}\right\rangle$ ) of the glycoproteins on the surface quantifies the capability of glycans in “replacing” the flexibility of a proteinaceous fragment close to the contact region at the glycoprotein–surface interface. In previous work on the open RBDs^8^ we showed that the protein’s flexibility at the interface can promote the number of contacts by increasing the deformation of the glycoprotein upon adsorption. The role of the glycan in the open RBD adsorption was really minor. However, in the closed conformation, the glycan drastically influences the whole interaction at the RBD–surface interface by amplifying or reducing the effects of flexibility in different protein contact regions. In particular, we show that, upon adsorption, the RBM contact region is up to 50% more flattened (SI Figure S1) than the head one. Hence, the glycan can dominate adsorption by gluing to the RBM contact region and blocking those specific short-range interactions found at the RBM region and effectively reducing the number of contacts at the glycoprotein–surface interface. Interestingly, mutations are another parameter that also plays a key role in the deformation upon adsorption because depending on the character of the residues, they will selectively and/or collectively increase hydrophobic or hydrophilic interactions with each of the model surfaces. We expect that the selective initial conformation of glycans toward the protein wall could be experimentally controlled by the surface, as shown by adsorption studies onto different surfaces.^34^ In addition, the local electrostatic properties of each monosaccharide of the glycan sequence could be exploited together with the protein mutations to tune the glycan–protein interaction.

Our results, based on the initial position of the glycan in either closed-head or closed-RBM regions, also provide evidence of clearly distinctive behaviors, which we discuss in the following paragraphs along with the evaluated interfacial properties. The computed contact area in the closed-head conformation doubles that of the closed-RBM for the hydrophilic surfaces in all VoCs. We calculate the number of contacts between the glycoprotein–surface and display them as the footprints of the top 5 residues with the glycan. This analysis enables us to interpret the glycan interaction preferences under different scenarios, such as the glycan located between the RBD-protein and the planar hydrophobic or hydrophilic surfaces. Based on our analysis, we identify three archetypal behaviors of the glycan upon adsorption: tethered-adsorption, repulsive squeeze-out, and soft-tethering (intermittent surface anchoring). These opposing behaviors can be combined dynamically during adsorption, depending on the precise glycan environment. For instance, mutations in the Omicron RBD-head favor hydrophobic interactions where the glycan squeezes out from the “panini” formed between the protein and the hydrophobic surface. Consecutively, the glycan enters into a new interaction-context at the liquid-surface interface, identified as soft-tethering. Similarly, the WT glycan in the closed-RBM conformation is able to tether the hydrophobic surface and find strong hydrophobic interactions that can hold the rest of the glycoprotein during desorption processes, exemplifying tethered-adsorption. Similar observations are currently discussed in the literature for glycan–membrane interactions^35,36^ and glycan–glycan or glycan–protein contexts.^13,37^ However, to the best of our knowledge, our work is pioneering in the characterization of glycan properties in “2D” during adsorption processes applicable to glycoprotein–fomite interfaces. This delineates a significant route for the molecular understanding of airborne transmission of disease, e.g., droplets landing on ubiquitous material surfaces, such as commonly used plastics or metals. Furthermore, we quantify the 2D footprints of the protein and glycan residues with the highest RMSFs, which allow us to identify whether fluctuations promote more contacts at the interface or whether those contacts are rather originated on highly localized short-range interactions within less flexible protein regions. For example, WT in the closed-RBM conformation has 3 common residues between the highest-contacts and highest-fluctuations at the interface. Remarkably, the same RBD-glycan shows high flexibility and the largest fluctuation areas, reaching 2 orders of magnitude greater values than those of the highest-fluctuating protein residues. The glycan’s increase in fluctuation areas originates in the squeeze-out and soft-tethering mechanisms described previously. The amphipathic character of the RBD’s glycan is also analyzed conveniently with our model surface setup. Here, we can prove that for this specific and globally neutral glycan, its hydrophobic number of contacts is consistently higher than the hydrophilic ones toward their respective surfaces. This was also confirmed by computing the angles of the sugar rings with respect to the surface normal.

Our models of single-RBDs^8^ are validated by whole trimer simulations, covering the spike ectodomain glycoprotein sequences detailed in Figure 10 with reused structural models for the spike ectodomain originating in WT^1^ and Omicron^38^ and shared within the computational biophysical community.^39^ The systematic upscaling study successfully validates the extended simulations of the single-RBD system with the spike ectodomain glycoproteins by strikingly matching over half of the interactions for the closed-head and closed-RBM initial conformations.

**Figure 10:**
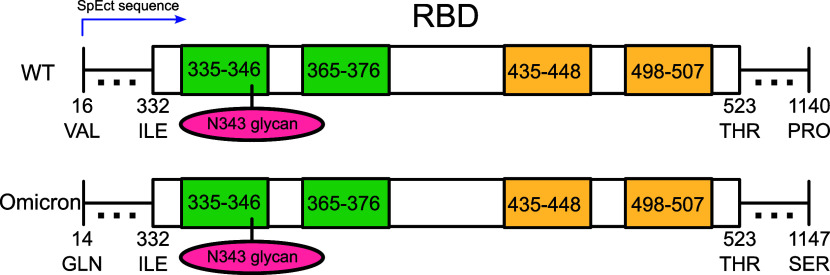
Schematic of residues taken into account for the analysis of the RBDs in the Spike and the simulated domain of the Spike SpEct glycoprotein for WT (top) and Omicron (bottom). Green (Head region) and orange (contact-to-surface RBM) regions highlight the contact regions that are considered for the isolated-RBD, consistent with SI Tables S1 and S2, summing the residue indexes by 331. Note that the FA2G0 N343 glycan is shown to be bound to one of the contact regions.

In a nutshell, we present different landing footprints and deformation mechanisms of the RBD glycoproteins, which can be directly applied to understand the influence of the glycans on their adsorption. Beyond providing molecular understanding of the complex interplay between molecules of different flexibilities, namely, protein fragments and glycans, this work offers a systematic method to analyze glycoproteins’ short-range interactions, which, at the same time, can provide proper molecular interpretation of adsorption experiments with high-resolution experimental techniques.^11,25,29^

Identifying novel adsorption mechanisms between glycoproteins and convenient model surfaces, together with the very recent development of experimental techniques tracking glycans with angstrom resolution^29^ among others, provides the starting point and momentum for the molecular understanding of these processes at heterogeneous interfaces, such as fomites, and systematically examines applied systems, namely, protein aggregation, multiprotein assemblies, misrecognition of proteins, and surfaces functionalized with glycans.

## Methods

### Structural Models

#### Polarizable Bilayer Model

The hydrophobic (PBL0) and hydrophilic (PBL1) surfaces were built from a small patch of decanol (DOL) molecules, where the OH– groups of the DOL chains are tuned to have 0 or 1 polarity for hydrophobic and hydrophilic surfaces (see SI Figure S18), respectively.^22^ To conserve the shape of the bilayer and prevent any effects on the mechanical properties of the surfaces, we added restraints to the two carbon atoms in the alkane chain, as discussed in previous works.^22,40,41^ Thus, the bilayer model does not include any molecular defects and does not show any curvature. We used *gmx editconf* to replicate and adjust the size of the DOL slabs.

#### Receptor-Binding Domain (RBD) Model

All RBD models were designed with the same protocol as in our previous paper^8^ where the RBD proteins are adopted from Barroso da Silva and coworkers,^30^ in which we added disulfide bonds to the VoCs and used Glycan Modeler from CHARMM-GUI to incorporate the FA2 glycan. The RBD models for WT, Delta, and Omicron were added to cubic boxes (see Table 3) containing polarized bilayers. For each isolated RBD simulation, we determined the contacts between the RBD and the SpEct region. Position restraints of 250 kJ mol^–1^ nm^–2^ were applied to those contacts in *x* and *y* axes to mimic the presence of the remaining SpEct glycoprotein, while enabling adsorption and desorption by keeping the *z*-axis free of restraints in all RBD-SpEct contacts (see in SI Figure  S19 for a graphical representation of restraint positions). All other residues not in contact with the SpEct were considered as flexible regions of the RBD. It must be noted that all structural analyses focused on the regions of the proteins in contact with the model surfaces. These residues are listed in SI Tables S1 and S2. No positional restraints were added to residues in the region in contact with the model surfaces. Note that residues in isolated RBDs were reindexed to 1 with respect to the spike ectodomain protein. Indexes can be related by summing 331 to the residue index in the isolated RBD. This procedure was repeated for both polarities, with three replicas per VoC (WT, Delta, and Omicron) and 2 glycan configurations relative to the RBD, resulting in a total of 36 configurations for RBD-PBLs with glycans. For each of these configurations, we performed 300 -ns-long simulations. Finally, simulations of RBD-PBLs in open configurations from our previous work^8^ were used as a reference to compare with our new results.

**Table 3:** Detailed Configurations of the Simulated Systems^a^

	VoC	No. of replicas	Box size	Atoms	Time (ns)
RBD	WT	3	7.5 × 7.5 × 12	2980	300
	Delta	3	7.5 × 7.5 × 12	2993	300
	Omicron	3	7.5 × 7.5 × 12	3036	300
	WT + Glyc	6 (3 head + 3 RBM)	8.6 × 7.6 × 12	2979 + 192	300
	Delta + Glyc	6 (3 head + 3 RBM)	8.6 × 7.6 × 12	2992 + 192	300
	Omicron + Glyc	6 (3 head + 3 RBM)	8.6 × 7.6 × 12	3035 + 192	300
Spike	WT	3	16.9 × 22.5 × 25.0	63793	300
	Omicron	3	16.9 × 22.5 × 25.0	64339	300

a Note that 848 decanol molecules, for a total of ∼78k atoms with water and ions, were used in simulations of the isolated RBDs in the presence and absence of glycans, and 5028 decanol molecules, ∼950k total atoms with water and ions, were used in simulations of the whole SpEct glycoprotein. Simulations of RBD-PBLs with glycans are performed in two different configurations of the glycans relative to the RBD: a glycan in the head–surface interface (head) and an RBM–surface interface (RBM) configuration. Representations of the initial configurations are shown in Figure 1. Details of simulations used for the open-RBDs are shown in our previous work.^8^

#### Spike Ectodomain Model (SpEct)

Spike ectodomain glycoprotein models were used from Casalino et al.^1^ for the WT variant, which is a glycosylated model of PDB ID 6VSB, and the glycosylated model of Omicron BA.1 (B.1.1.529) was used from Kim et al.^38^ based on PDB ID 7TEI. For the Spike-PBL simulations, mechanical restraints were not added to simulations of the entire spike ectodomain glycoprotein, as it is bound to the highly flexible S2 membrane protein, which is simultaneously shown to be highly diffusive over the envelope.^10,34,42,43^ Production simulations of three replicas were carried out, where the SpEct glycoprotein was set to rotations of up to 3° from the reference replica. It must be noted that, despite simulating the whole SpEct glycoprotein, analyses were focused only on the RBDs, ensuring the same sequence as our previously described isolated RBD simulations (see Figure 10).

### Molecular Dynamics

For this study, we computed simulations of different scales of the system: the receptor-binding domains and the spike ectodomain protein of SARS-CoV-2 for three variants of concern (VoCs). All-atom simulations were carried out with Gromacs 2023,^44^ and the system components (protein, ions, and the polarizable bilayer) were modeled using CHARMM36 ^45,46^ force field and TIP3P^47^ for the water. Ions were added only to neutralize the system. CHARMM-GUI’s Glycan Modeler was used to join the glycan to the RBDs. Periodic boundary conditions were applied, and PME was used for long-range electrostatics with *r*_coulomb_ = *rnlist* = 1.4 Å and a Fourier spacing of 0.175 (optimal value is *r*_coulomb_/8). Minimization was done by steepest descent (50 000 steps) with an initial step size of 0.01 nm and a maximum force tolerance of 1000 kJ/(mol nm). Thermalization (NVT) and equilibration (NPT) of the system were carried out for 100 ps at 300 K and 1 bar pressure, with an integration step of 2 fs. Production runs were performed under the same conditions as equilibration and thermalization for 300 ns. Energy minimization used CPUs, while all production runs used 1xGPU, as the former scaled better than 2xGPUs for our systems.

Finally, 300 ns of trajectories for each replica were collected for all of the systems summarized in Table 3. Starting configurations from the MD production can be found in the Zenodo repository listed below (Table 4).

**Table 4:** Number of Replicas per Variant and Surface Polarity Used for Each Figure in the Manuscript^a^

Figure id	Closed RBD	Closed RBD w glyc	Open RBD	Open RBD w glyc	SpEct OCC
2	-	3	3	-	-
3	3	3	3	-	-
4	1	1	-	1	-
5	1	1	-	1	-
6	1	1	-	1	-
7	-	3	-	-	-
8	-	3	-	-	-
9	-	-	-	-	1

a That is, for instance, in Figure 9, one SpEct glycoprotein replica for WT and Omicron variants was made for each of the two polarized bilayers. Note that despite only using one replica in SpEct simulations for Figure 9, three replicas were used for the glycan contact context in SI Table S11. Replica id used for figures with a single replica is listed in SI Table S12.

### Analysis

Our analyses were carried out using our previously published MDAnalysis-based toolkit 2DAnalysis,^23^ core MDAnalysis functions, and in-GROMACS tools such as SASA computation. It must be noted that only the contact regions of the proteic RBDs (listed in Tables S1 and S2) were considered for analyses and not the whole protein.

#### Parallel and Perpendicular Radii of Gyration

The parallel and perpendicular radii of gyration give structural information about the adsorption phenomenology. $R_{g∥}$ gives information on how the protein is stretched parallel to the surface, and $R_{g⊥}$ offers a measure of how the biopolymer corrugates or flattens perpendicular to the surface. They are computed as:

1 $$R_{g}=\sqrt{\frac{1}{m_{T}}\underset{i}{\sum }m_{i}\left[{\left(x_{i}-x_{\mathrm{CM}}\right)}^{2}+{\left(y_{i}-y_{\mathrm{CM}}\right)}^{2}+{\left(z_{i}-z_{\mathrm{CM}}\right)}^{2}\right]}$$

2 $$R_{g∥}=\sqrt{\frac{1}{m_{T}}\underset{i}{\sum }m_{i}\left[{\left(x_{i}-x_{\mathrm{CM}}\right)}^{2}+{\left(y_{i}-y_{\mathrm{CM}}\right)}^{2}\right]}$$

3 $$R_{g⊥}=\sqrt{\frac{1}{m_{T}}\underset{i}{\sum }m_{i}{\left(z_{i}-z_{\mathrm{CM}}\right)}^{2}}$$

where **R**_CM_ = (*x*_CM_, *y*_CM_, *z*_CM_) is the position of the center of mass, *m*_*i*_ is the mass of each residue, and *m*_T_ is the total mass of the residues.

Similarly to previous works, we have computed the $\left\langle R_{g⊥}^{2}\right\rangle /\left\langle R_{g∥}^{2}\right\rangle$ ratios to characterize the flexibility of the glycan and the contact region of the protein, where the ratio value at values under 0.32 is indicative of a semiflexible regime in which the flexibility is mainly driven in a parallel-to-surface fashion.^19–21^ This analysis was carried out using the 2DAnalysis toolkit.^23^

#### Total Contacts vs Contact Areas

Total contacts of the RBDs with the surface were computed by measuring the distance of each residue of the RBD with each OH– group in the decanol residues and counting as contacts all those with distances under 14 Å. On the other hand, the contact areas were computed by calculating the SASA of the protein, glycan, and surface separately, as well as as a whole, and following (SASA_BioPol_ + SASA_PBL_ – SASA_BioPol + PB_)/2, where BioPol represents the RBD, the glycan or RBD + Glycan. SASA calculations were carried out by the GROMACS SASA algorithm.

#### Density Contour Plots

Density contour plots were generated with the 2DAnalysis toolkit^23^ and applied to footprint residue figures (Figures 4 and 5; SI Figures S2, S3, S8 and S9) and hydrogen-bond figures (Figures 6 and SI Figure S10) to map the positioning of the protein and the glycan during simulations. Only residues within contact regions and frames where the contact regions of the protein were within 14  Å of decanol oxygen atoms were included, excluding desorbed frames.

For Figure 5, and SI Figures S8 and S9, residue areas were obtained by integrating the KDE contour densities of each residue during the simulation using the getArea function in 2DAnalysis. In these cases, the contour densities of residues were computed considering the 1500 (300) frames with the most contact of the protein’s contact region to the hydrophobic (hydrophilic) surface to ensure comparable areas for different replicas, variants, and glycan positioning.

#### Protein–Glycan Contact Maps

Contact maps were computed by measuring the distance between the centers of mass of each residue in the contact region of the protein and the glycan. All distances under 10 ñÅ between the residues of these two groups were considered a contact. SI Figures S12 and S13 show contact maps considering distances under 14 Å.

### Data and Software Availability

All-atom simulations were carried out with GROMACS 2023. The corresponding TPR files, and initial configuration in GRO format for MD production; videos of the trajectories, and the trajectories themselves without water in XTC format used in this work, and data from the analysis are available on the Zenodo repository (https://zenodo.org/records/17207608), and analysis scripts can be found in the GitHub repositories https://github.com/pyF4all/2DanalysisTutorials and https://github.com/ABoschF/Paper_Closed-RBDs_public.

## Supplementary Material



## Notes

Tables indicating the groups in each group, $\left\langle R_{g⊥}^{2}\right\rangle /\left\langle R_{g∥}^{2}\right\rangle$ ratios and their corresponding $\left\langle R_{g⊥}\right\rangle$ and $\left\langle R_{g∥}\right\rangle$ contributions, % H-bond during simulations, Glycan–protein contacts by residue types, secondary structure of contact regions, and replica ids used in analysis with only one replica used; and figures show the $\left\langle R_{g⊥}^{2}\right\rangle /\left\langle R_{g∥}^{2}\right\rangle$ per region, Contour plots with the footprint of top 5 contacts and top 5 RMSFs for Delta, and for WT and Omicron onto hydrophilic surfaces, representative snapshots of RBD interactions with the surfaces, violin plot of the relative angles of the sugar rings of the glycans with the normal of the surface, % H-bond during simulations for Delta, the contact maps for Delta with a 10 Å threshold, and WT, Delta, and Omicron with 14 Å, and snapshot of the context of glycan within the spike ectodomain glycoprotein. Details can be found in the Table of Contents of the SI (PDF)
