# Supplementary material for: Glycans Modulate the Adsorption of RBD Glycoproteins on Polarizable Surfaces
Source: J Chem Inf Model. 2026 Jan 15;66(5):2719–34. doi: 10.1021/acs.jcim.5c02363 (PMC12977037; doi:10.1021/acs.jcim.5c02363)
Supplement: Supplementary file 1 [file ci5c02363_si_002.pdf]

Supplementary Material: Glycans modulate the adsorption of RBD Glycoproteins on polarizable surfaces

Antonio M. Bosch-Fernández[o,†,‖] Willy Menacho,[‡,‖] Rubén Pérez,[*,†] and Horacio V. Guzman[o*,§,‖]

†*Departamento de Física Teórica de la Materia Condensada, Universidad Autónoma de Madrid, E-28049 Madrid, Spain*

‡*Institut de Ciència de Materials de Barcelona ICMAB-CSIC, Campus de la UAB, E-08193 Spain*

¶*Condensed Matter Physics Center (IFIMAC), Universidad Autónoma de Madrid, E-28049 Madrid, Spain*

§*Institut de Ciència de Materials de Barcelona ICMAB-CSIC, E-08193 Barcelona, Spain*

‖*Biophysics and Intelligent Matter Lab, E-08193 Barcelona, Spain*

E-mail: ruben.perez@uam.es; hguzman@csic.es

Table of Content

List of Supplementary Tables

- Table S1: Residues in Group 1 (RBD head) for WT, Delta, Omicron. 4
- Table S2: Residues in Group 2 (RBM contact-to-surface region). 5
- Table S3: Ratios of perpendicular/parallel Rg for contact region and glycan. 6
- Table S4: Mean perpendicular and parallel Rg values. 7
- Table S5: Hydrogen bonds in WT (open, closed-head, closed-RBM). 8
- Table S6: Hydrogen bonds in Omicron. 9
- Table S7: Hydrogen bonds in Delta. 10
- Table S8: Glycan–protein contacts by residue type (WT & Omicron, 14 Å). 11
- Table S9: Glycan–protein contacts by residue type (Delta, 10/14 Å). 11
- Table S10: Secondary structure percentages of contact regions. 12
- Table S11: Glycan contact context (14 Å cutoff). 12
- Table S12: Simulation replicas used for contour analyses. 13

List of Supplementary Figures

- Figure S1: Parallel and perpendicular Rg of contact regions (WT/Delta/Omicron). 14
- Figure S2: Contact footprints on hydrophilic surface (WT/Omicron). 15
- Figure S3: Contact footprints for Delta on hydrophobic/hydrophilic surfaces. 16
- Figure S4: Snapshots on hydrophobic surface. 17
- Figure S5: Snapshots on hydrophilic surface. 18
- Figure S6: Definition of glycan ring tilt angle θ_z. 19

- Figure S7: Violin/box plots of glycan tilt angles. 20
- Figure S8: RMSF footprints on hydrophilic surface (WT/Omicron). 21
- Figure S9: RMSF footprints for Delta on hydrophobic/hydrophilic surfaces. 22
- Figure S10: Surface hydrogen bonds for Delta (closed head/RBM, open). 23
- Figure S11: Histograms of glycan–surface contacts. 24
- Figure S12: Glycan–protein contact maps (WT/Omicron, 14 Å). 25
- Figure S13: Glycan–protein contact maps for Delta (10/14 Å). 26
- Figure S14: Context snapshots (WT, open). 27
- Figure S15: Context snapshots (Omicron, open). 27
- Figure S16: Context snapshots (WT, closed). 28
- Figure S17: Context snapshots (Omicron, closed). 28
- Figure S18: Diagram of the polarizable bilayer (PBL). 29
- Figure S19: Diagram of restraints in open vs closed RBD setups. 29

1 Supplementary Tables

Table S1: Table contains the ResIDs and corresponding residue names of residues used in Group 1 (Head of the RBD). Mutations are highlighted in fuchsia. **CN**: Charged Negative, **CP**: Charged Positive, **UP**: Uncharged Polar and **NP**: NonPolar

	ResIDs	WT	Delta	Omicron
1	4	LEU (NP)	LEU (NP)	LEU (NP)
2	5	CYS (NP)	CYS (NP)	CYS (NP)
3	6	PRO (NP)	PRO (NP)	PRO (NP)
4	7	PHE (NP)	PHE (NP)	PHE (NP)
5	8	GLY (NP)	GLY (NP)	**ASP (CN)**
6	9	GLU (CN)	GLU (CN)	GLU (CN)
7	10	VAL (NP)	VAL (NP)	VAL (NP)
8	11	PHE (NP)	PHE (NP)	PHE (NP)
9	12	ASN (UP)	ASN (UP)	ASN (UP)
10	13	ALA (NP)	ALA (NP)	ALA (NP)
11	14	THR (UP)	THR (UP)	THR (UP)
12	15	ARG (CP)	ARG (CP)	ARG (CP)
13	34	TYR (UP)	TYR (UP)	TYR (UP)
14	35	SER (UP)	SER (UP)	SER (UP)
15	36	VAL (NP)	VAL (NP)	VAL (NP)
16	37	LEU (NP)	LEU (NP)	LEU (NP)
17	38	TYR (UP)	TYR (UP)	TYR (UP)
18	39	ASN (UP)	ASN (UP)	ASN (UP)
19	40	SER (UP)	SER (UP)	**LEU (NP)**
20	41	ALA (NP)	ALA (NP)	ALA (NP)
21	42	SER (UP)	SER (UP)	**PRO (NP)**
22	43	PHE (NP)	PHE (NP)	PHE (NP)
23	44	SER (UP)	SER (UP)	**PHE (NP)**
24	45	THR (UP)	THR (UP)	THR (UP)

Table S2: Table contains the ResIDs and corresponding residue names of residues used in Group 2 (Contact-to-surface RBM region). Mutations are highlighted in fuchsia. **CN**: Charged Negative, **CP**: Charged Positive, **UP**: Uncharged Polar and **NP**: NonPolar

	ResIDs	WT	Delta	Omicron
1	104	ALA (NP)	ALA (NP)	ALA (NP)
2	105	TRP (NP)	TRP (NP)	TRP (NP)
3	106	ASN (UP)	ASN (UP)	ASN (UP)
4	107	SER (UP)	SER (UP)	SER (UP)
5	108	ASN (UP)	ASN (UP)	ASN (UP)
6	109	ASN (UP)	ASN (UP)	**LYS (CP)**
7	110	LEU (NP)	LEU (NP)	LEU (NP)
8	111	ASP (CN)	ASP (CN)	ASP (CN)
9	112	SER (UP)	SER (UP)	SER (UP)
10	113	LYS (CP)	LYS (CP)	LYS (CP)
11	114	VAL (NP)	VAL (NP)	VAL (NP)
12	115	GLY (NP)	GLY (NP)	**SER (UP)**
13	116	GLY (NP)	GLY (NP)	GLY (NP)
14	117	ASN (UP)	ASN (UP)	ASN (UP)
15	167	GLN (UP)	GLN (UP)	**ARG (CP)**
16	168	PRO (NP)	PRO (NP)	PRO (NP)
17	169	THR (UP)	THR (UP)	THR (UP)
18	170	ASN (UP)	ASN (UP)	**TYR (UP)**
19	171	GLY (NP)	GLY (NP)	GLY (NP)
20	172	VAL (NP)	VAL (NP)	VAL (NP)
21	173	GLY (NP)	GLY (NP)	GLY (NP)
22	174	TYR (UP)	TYR (UP)	**HIS (CP)**
23	175	GLN (UP)	GLN (UP)	GLN (UP)
24	176	PRO (NP)	PRO (NP)	PRO (NP)

Table S3: $\langle Rg_{\perp}^2 \rangle / \langle Rg_{\parallel}^2 \rangle$ radii of gyration ratios of the contact region of the closed and open RBDs, and the glycan during the simulations of the closed RBDs with the two glycan-RBD conformations for the three VoCs. These values are represented as symbols in Figure 2. Ratio values under 0.32 are considered to be in the polymer semiflexible regime.[1] Open does not have closed/head or closed-RBM configurations; its contact region is only proteic.[2]

	Glycan	Variant	$\langle Rg^2_{closed,\perp} \rangle / \langle Rg^2_{closed,\parallel} \rangle$	$\langle Rg^2_{glycan,\perp} \rangle / \langle Rg^2_{glycan,\parallel} \rangle$	$\langle Rg^2_{open,\perp} \rangle / \langle Rg^2_{open,\parallel} \rangle$
PBL0	head	WT	0.071	0.087	0.060
		Delta	0.081	0.119	0.057
		Omicron	0.065	0.130	0.064
	RBM	WT	0.105	0.149	-
		Delta	0.080	0.060	-
		Omicron	0.072	0.183	-
PBL1	head	WT	0.070	0.385	0.060
		Delta	0.074	0.258	0.057
		Omicron	0.077	0.205	0.060
	RBM	WT	0.103	0.445	-
		Delta	0.079	0.368	-
		Omicron	0.070	0.318	-

Table S4: Mean perpendicular and parallel radii of gyration of the contact region of the closed and open RBDs, and the glycan during the simulations of the closed RBDs with the two glycan-RBD conformations for the three VoCs. These values are represented as symbols in Figure 2. Open does not have closed/head or closed-RBM configurations; its contact region is only proteic.[2]

		Variant	$Rg_{\perp}^{closed}$	$Rg_{\parallel}^{closed}$	$Rg_{\perp}^{glyc}$	$Rg_{\parallel}^{glyc}$	$Rg_{\perp}^{open}$	$Rg_{\parallel}^{open}$
PBL0	head	WT	3.43	12.87	2.23	7.71	3.64	14.85
		Delta	3.83	13.46	2.50	7.37	3.59	15.04
		Omicron	3.44	13.47	2.51	7.45	3.84	15.14
	RBM -	WT	4.12	12.72	2.64	7.09	-	-
		Delta	3.80	13.48	1.87	7.85	-	-
		Omicron	3.65	13.58	2.82	7.10	-	-
PBL1	head	WT	3.43	12.95	4.02	6.66	3.59	14.70
		Delta	3.66	13.51	3.34	6.91	3.58	15.04
		Omicron	3.63	13.10	3.01	7.07	3.74	15.30
	RBM	WT	4.08	12.73	4.10	6.33	-	-
		Delta	3.74	13.37	3.95	6.66	-	-
		Omicron	3.55	13.39	3.59	6.78	-	-

Table S5: Residues involved in hydrogen bonding during simulations of the Wild-Type (WT) variant in three configurations: open RBD, closed with the glycan positioned between the RBD head and the surface, and closed with the glycan between the RBM and the surface (top to bottom, respectively). The table reports the residue IDs, names, and the percentage of simulation time in which each residue participated in hydrogen bonding. Top five residues are represented in Fig. 6.

	ResIDs	ResNames	% Hbonds
WT Glycan in Head	197	AMAN	36.24
	199	AMAN	35.80
	198	BGLCNA	30.64
	200	BGLCNA	22.40
	194	AFUC	9.64
	196	BMAN	6.44
	195	BGLCNA	4.56
	109	ASN	3.16
	39	ASN	0.13
	38	TYR	0.02
WT Glycan in RBM	199	AMAN	17.42
	200	BGLCNA	14.84
	197	AMAN	14.07
	198	BGLCNA	10.04
	39	ASN	7.53
	195	BGLCNA	5.64
	196	BMAN	2.49
	38	TYR	2.31
	35	SER	0.91
	194	AFUC	0.78
	42	SER	0.04
WT OPEN	169	THR	54.67
	167	GLN	17.90
	153	GLU	11.20
	115	GLY	1.63
	118	TYR	0.83
	170	ASN	0.60
	116	GLY	0.57
	147	THR	0.43
	117	ASN	0.33
	150	ASN	0.27
	156	ASN	0.07
	146	SER	0.03

Table S6: Residues involved in hydrogen bonding during simulations of the Omicron variant in three configurations: open RBD, closed with the glycan positioned between the RBD head and the surface, and closed with the glycan between the RBM and the surface (top to bottom, respectively). The table reports the residue IDs, names, and the percentage of simulation time in which each residue participated in hydrogen bonding. Top five residues are represented in Figure 6.

	ResIDs	ResNames	% Hbonds
Omicron Glycan in Head	194	AFUC	54.31
	199	AMAN	48.40
	109	LYS	35.00
	197	AMAN	29.98
	200	BGLCNA	27.16
	39	ASN	20.58
	198	BGLCNA	20.58
	196	BMAN	14.27
	195	BGLCNA	8.67
	193	BGLCNA	7.89
	108	ASN	1.33
	169	THR	0.16
	38	TYR	0.04
Omicron Glycan in RBM	200	BGLCNA	60.62
	199	AMAN	44.44
	197	AMAN	41.71
	196	BMAN	39.53
	39	ASN	17.73
	194	AFUC	15.53
	195	BGLCNA	10.67
	198	BGLCNA	8.09
	38	TYR	0.87
	109	LYS	0.16
Omicron Open RBD	170	TYR	48.97
	167	ARG	32.73
	169	THR	14.53
	115	SER	13.80
	150	ASN	7.97
	156	ASN	5.60
	118	TYR	5.40
	147	LYS	0.70
	158	TYR	0.30
	154	GLY	0.23
	151	GLY	0.17

Table S7: Residues involved in hydrogen bonding during simulations of the Delta variant in three configurations: open RBD, closed with the glycan positioned between the RBD head and the surface, and closed with the glycan between the RBM and the surface (top to bottom, respectively). The table reports the residue IDs, names, and the percentage of simulation time in which each residue participated in hydrogen bonding. Top five residues are represented in Figure S10.

	ResIDs	ResNames	% Hbonds
Delta Glycan in Head	197	AMAN	42.02
	199	AMAN	35.47
	194	AFUC	32.82
	198	BGLCNA	31.62
	200	BGLCNA	29.07
	196	BMAN	11.33
	109	ASN	9.93
	195	BGLCNA	0.49
	113	LYS	0.42
	39	ASN	0.09
	108	ASN	0.07
	169	THR	0.02
Delta Glycan in RBM	200	BGLCNA	43.40
	197	AMAN	29.60
	199	AMAN	28.87
	198	BGLCNA	19.27
	196	BMAN	3.96
	194	AFUC	2.00
	195	BGLCNA	1.51
	39	ASN	0.24
	109	ASN	0.20
	113	LYS	0.02
Delta OPEN	153	GLU	46.80
	167	GLN	15.63
	115	GLY	10.93
	169	THR	4.47
	147	LYS	3.70
	118	TYR	3.27
	150	ASN	0.90

Table S8: Contact frequencies between protein residues and the glycan, grouped by residue type (positively charged, negatively charged, non-polar, and uncharged polar) for WT and Omicron with a 14Å contact cutoff. Values are normalized by the total number of glycan residues, and the number of simulation frames considered. A value equal to the number of residue in a given type (in parenthesis) corresponds to the theoretical case where there is at least one contact of glycan residue with one protein residue. Note that glycan residue can have more than one contact with protein residues. Glycines have been excluded from the count due to their low polarity.

		Variant	Positive	Negative	Non-Polar	Uncharged Polar
head	PBL0	WT	0.062 (2)	2.339 (2)	2.964 (18)	1.180 (21)
		Omicron	0.168 (4)	4.121 (3)	2.081 (21)	0.857 (16)
	PBL1	WT	0.147	2.081	2.313	1.398
		Omicron	0.173	3.148	2.298	1.143
RBM	PBL0	WT	4.130	3.742	1.628	1.014
		Omicron	2.910	3.034	1.763	1.924
	PBL1	WT	3.954	3.429	1.847	1.489
		Omicron	2.982	3.240	2.174	2.255

Table S9: Contact frequencies between protein residues and the glycan, grouped by residue type (positively charged, negatively charged, non-polar, and uncharged polar) for Delta with 10Å and(|) 14Å cutoff, analogue treatment to Tables 1 and S8. In parentheses, the number of residues of the corresponding residue type, and glycines have been excluded from the count due to their low polarity.

	Surface	Variant	Positive	Negative	Non-Polar	Uncharged Polar
head	PBL0 (hydrophobic)	Delta	0.000|0.001	0.429|1.583	0.915|2.636	0.463|1.443
	PBL1 (hydrophilic)	Delta	0.000|0.052	0.264|1.068	0.651|2.104	0.596|1.653
RBM	PBL0 (hydrophobic)	Delta	1.740|4.147	1.366|3.526	0.547|1.485	0.607|1.742
	PBL1 (hydrophilic)	Delta	0.316|1.401	0.538|2.059	0.570|1.627	0.555|1.616

Table S10: Percentage of secondary structure (%SecStruc) of contact regions of the RBDs in bulk during short 10ns simulations. Here, we can evidence a difference of at least 15% more coils in the closed RBD contact region respect to the open configurations in absence of surface.

	Region	Coil	Helix	Sheet
WT	open	74.93	6.80	18.28
	closed	56.95	28.77	14.27
Delta	open	77.42	4.61	17.97
	closed	57.97	27.94	14.09
Omicron	open	77.42	4.17	18.41
	closed	60.79	26.44	12.77

Table S11: Glycan interactions for SpEct glycoprotein–PBLs simulations. Table reports the mean contact occurrence of glycans during the simulations with a 14Å contact-cutoff. "Glycan" label refers to the Glycan in the corresponding RBD, "Other Glycans" refers to all glycans but the glycan in the corresponding RBD, and "remaining SpEct" refers to all the protein residues in the Spike ectodomain protein, but those in the RBD to which it is attached.

RBD type	Glycan w/ RBD's head	Other Glycans w/ RBD's head	Glycan w/ Other Glycans
WT Open	63.661	18.467	0.440
Omicron Open	68.553	11.573	8.766
WT Closed	60.149	13.914	0.435
Omicron Closed	54.166	16.313	3.438
RBD type	Glycan w/ RBD's RBM	Other Glycan w/ RBD's RBM	Glycan w/ remaining SpEct
WT Open	14.375	0.546	17.523
Omicron Open	9.138	0.550	7.566
WT Closed	7.595	10.625	35.138
Omicron Closed	10.931	16.517	16.414

Table S12: Replica id used for the analysis with contour plots (Figures 4, 5, S8, S3, and S9), with the exceptions of H-bond analysis that considers all three replicas. Open-RBD trajectories are the same as those used in our previous work[2] in the presence of glycans.

	closed-head	closed-RBM	Spike UDD
WT-PBL0	1	1	1
Delta-PBL0	2	2	-
Omicron-PBL0	1	3	2
WT-PBL1	1	3	1
Delta-PBL1	2	2	-
Omicron-PBL1	3	2	2

2 Supplementary Figures

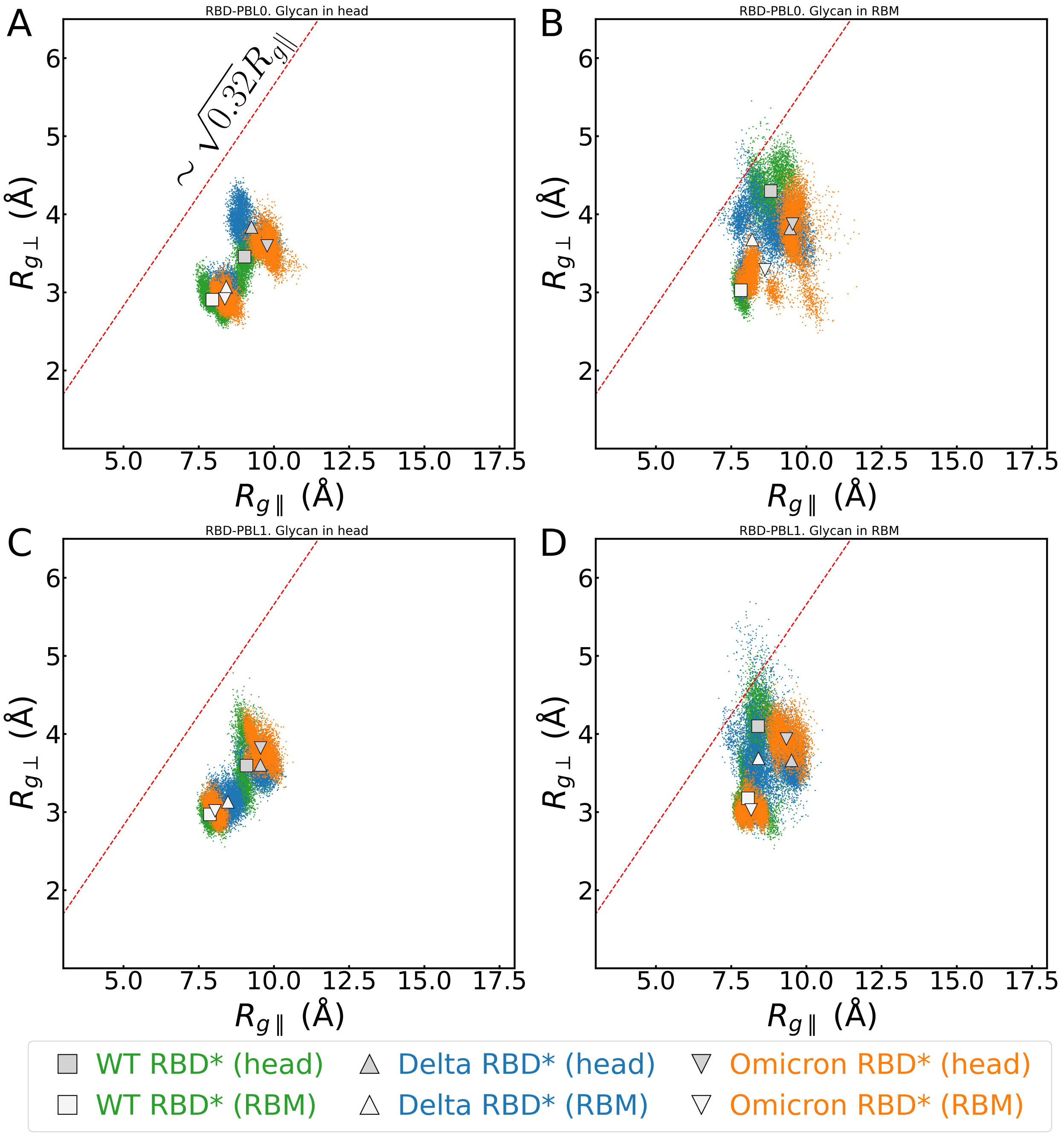

Figure S1: Parallel and perpendicular radii of gyration of the contact region of the head- and RBM-RBDs for WT (squares, green), Delta (triangles, orange), and Omicron (inverted triangles, blue) variants. Note that these are the contributions per region of white-filled colored symbols in Figure 2. The Red lines indicate the limit $\sim \sqrt{0.32} R_{g\parallel}$ for the semiflexible polymer regime. Panels show closed-RBD with the glycan between (A) a hydrophobic surface and the RBD's head, (B) a hydrophobic surface and the RBD's RBM, (C) a hydrophilic surface and the RBD's head, and (D) a hydrophilic surface and the RBD's RBM.

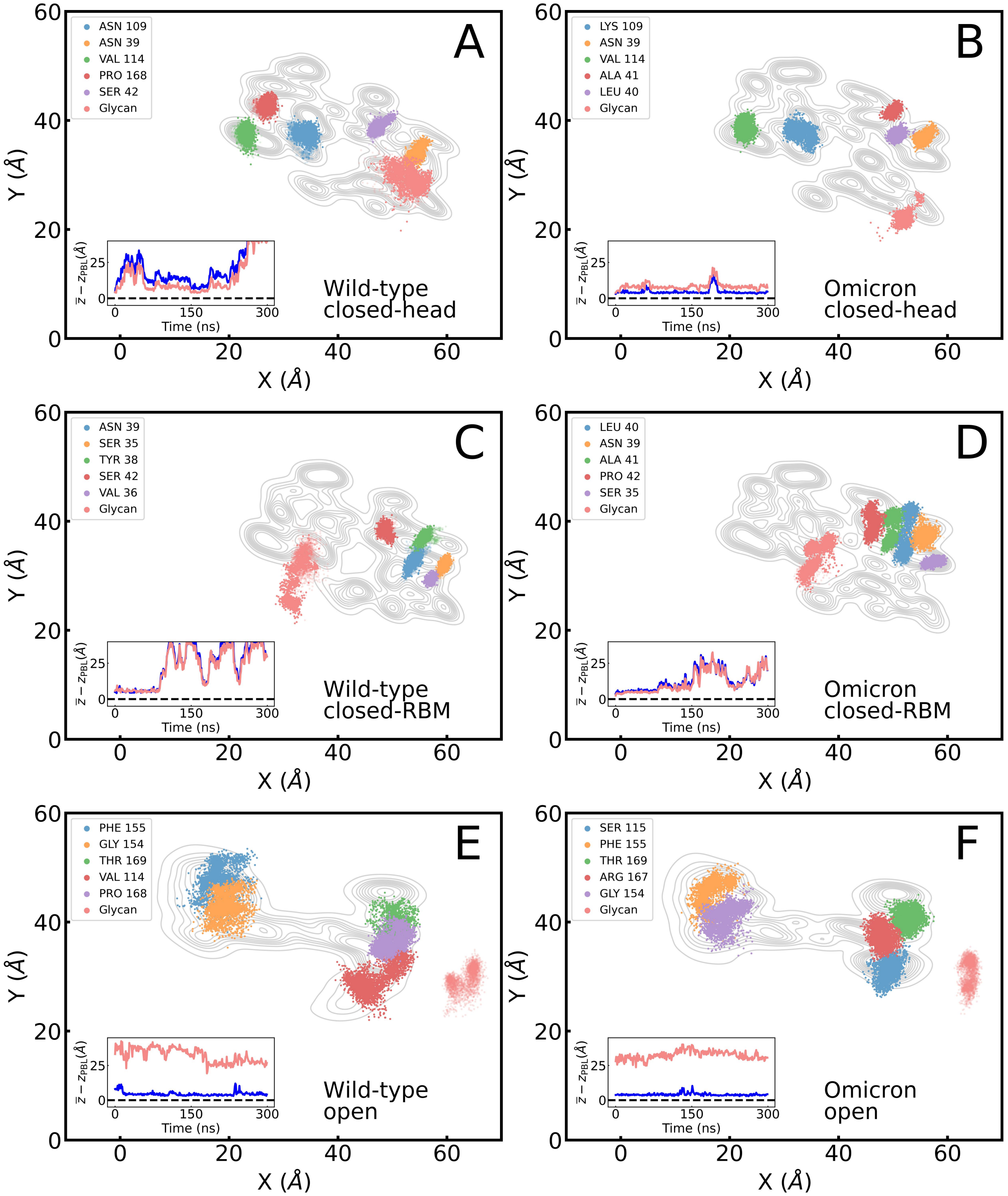

Figure S2: Footprint of the top five protein residues with the highest number of contacts to the hydrophilic surface and center-of-mass (COM) of the glycan for the closed- and open-RBD configurations for WT (left column) and Omicron variants (right column). Insets show the average distance of these residues (blue) and the glycan (red) to the surface. Panels display results for (A, B) closed-RBD with the glycan between the RBD head and the surface, (C, D) closed-RBD with the glycan between the RBM and the surface, and (E, F) open-RBD configuration. Contours of the contact regions of closed and open RBDs are shown as a reference map for the protein.

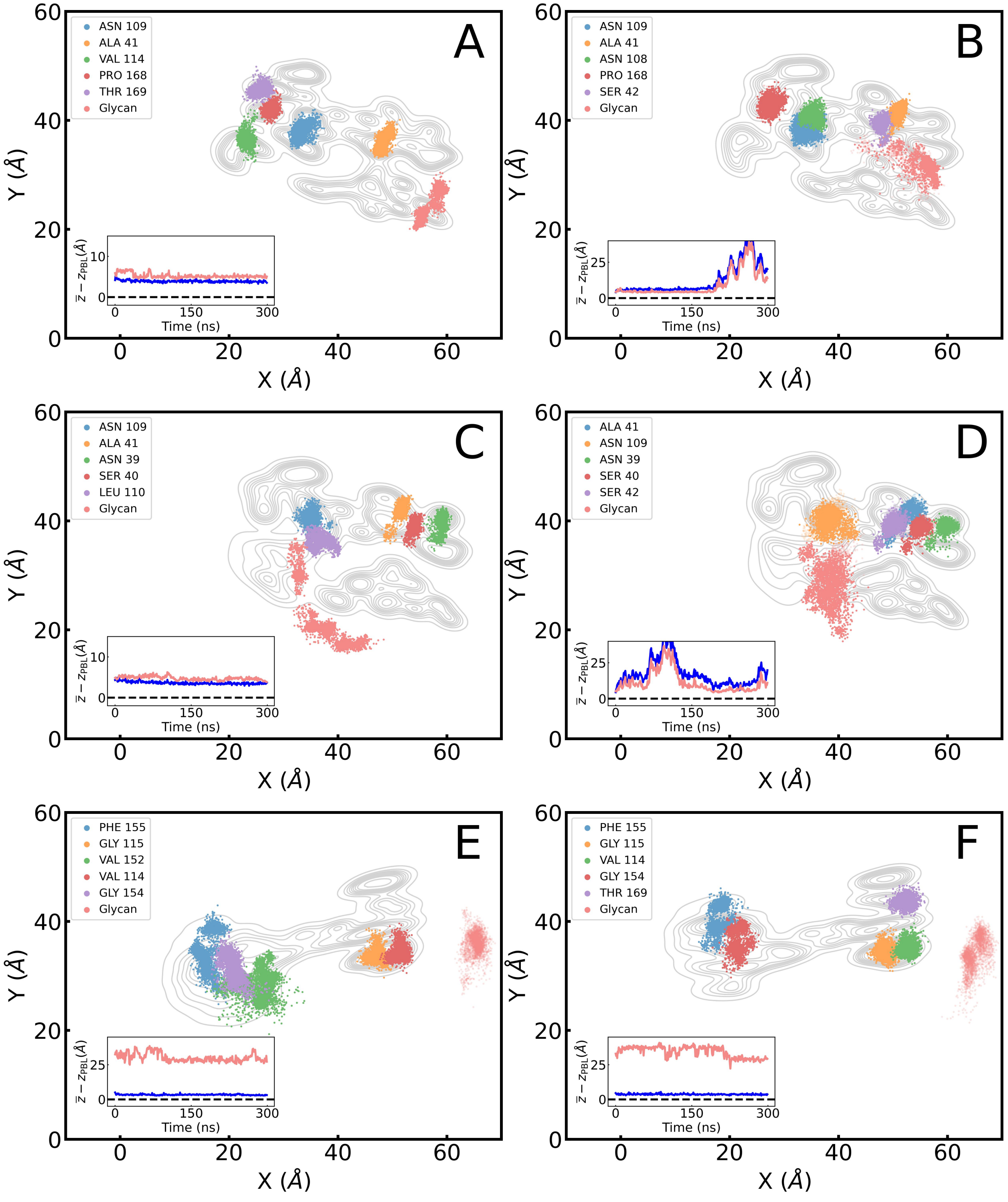

Figure S3: Footprint of the top five protein residues with the highest number of contacts to the hydrophobic (left column) and hydrophilic (right column) surfaces, and center-of-mass (COM) of the glycan for the closed- and open-RBD configurations for Delta variant. Insets show the average distance of these residues (blue) and the glycan (red) to the surface. Panels display results for (A, B) closed-RBD with the glycan between the RBD head and the surface, (C, D) closed-RBD with the glycan between the RBM and the surface, and (E, F) open-RBD configuration. Contours of the contact regions of closed and open RBDs are shown as a reference map for the protein.

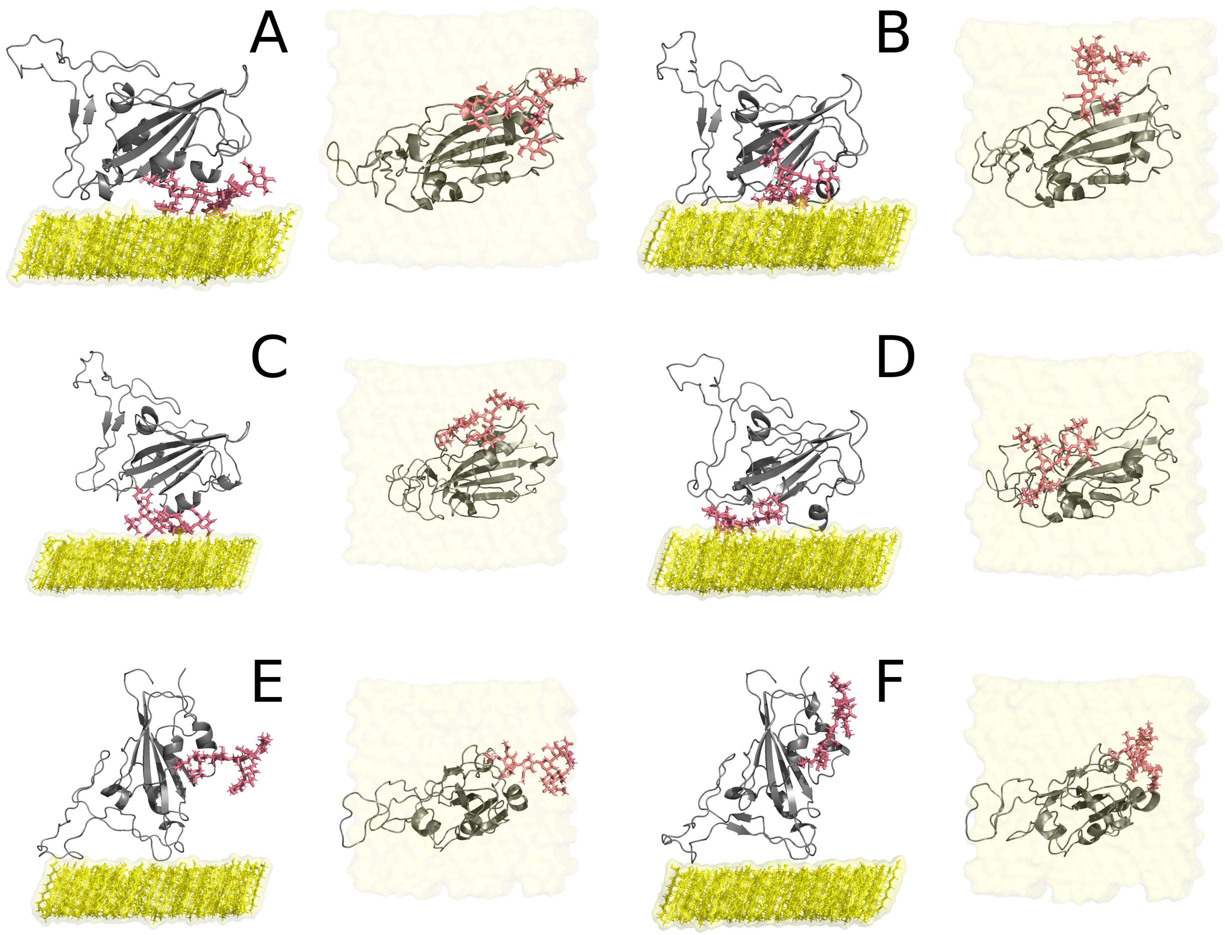

Figure S4: Snapshots of the RBDs with glycans onto the hydrophobic surface at 150ns, with the exception of (C) at 100ns, to evidence the anchoring of the protein via glycan. Replicas and panel ordering are consistent to simulations with the footprint contour plots (Figure 4 and 5.

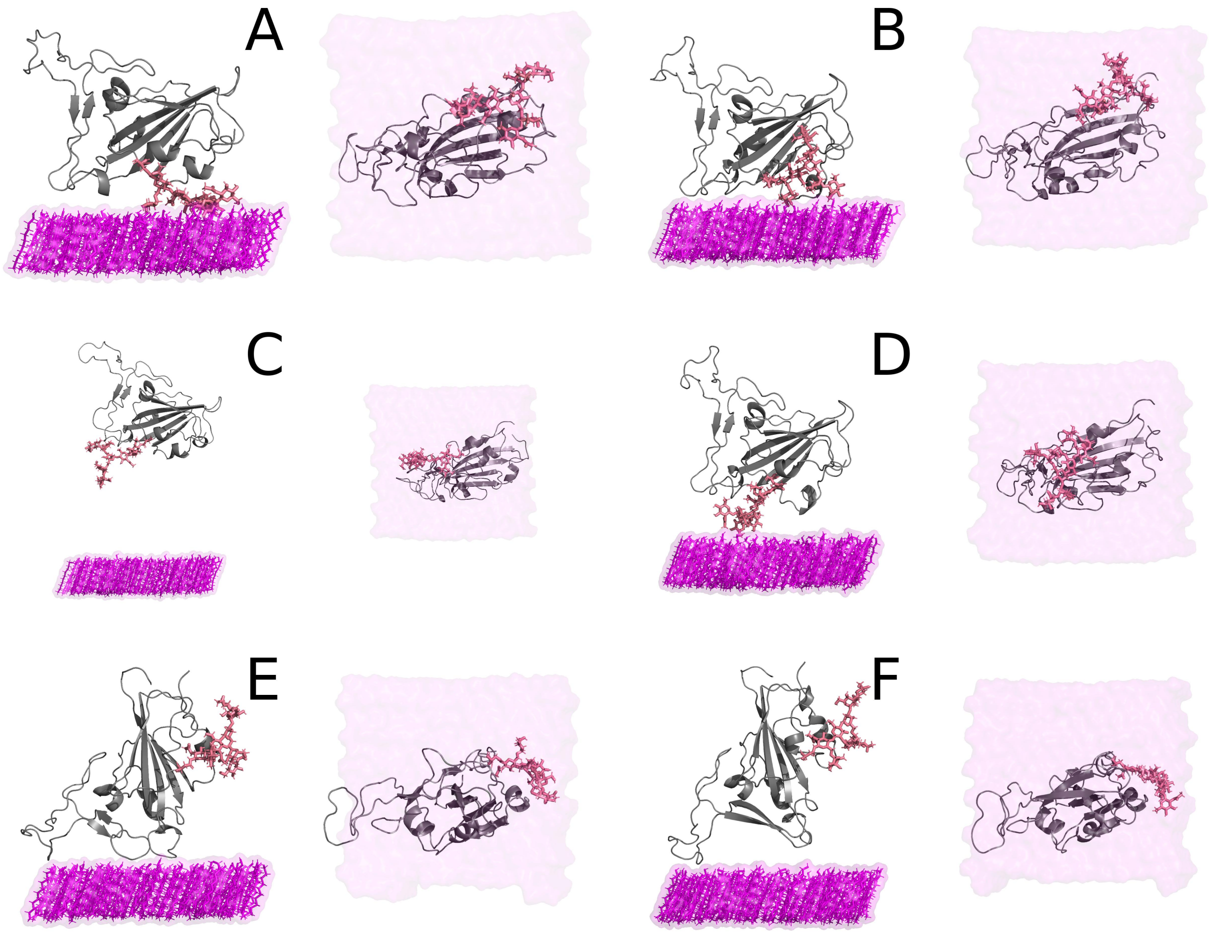

Figure S5: Snapshots of the RBDs with glycans onto the hydrophilic surface at 150ns. Replicas and panel ordering are consistent to simulations with the footprint contour plots (Figure S2 and S8).

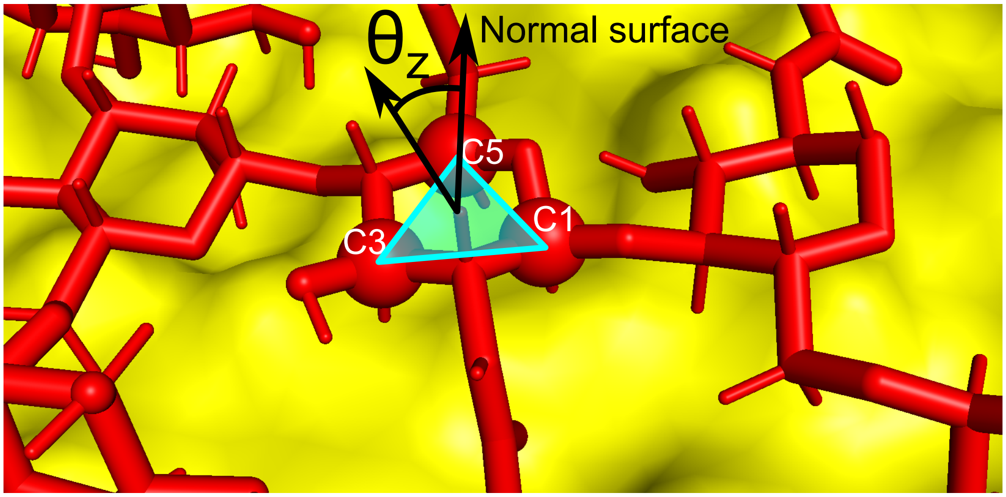

Figure S6: Description of the computed angles in Figure S7. θ_z is defined as the angles between the normal plane of the monosaccharide ring and the z-axis (plane normal). Note that the monosaccharide ring plane is defined by the position of the C1, C3 and C5 atoms of the ring.

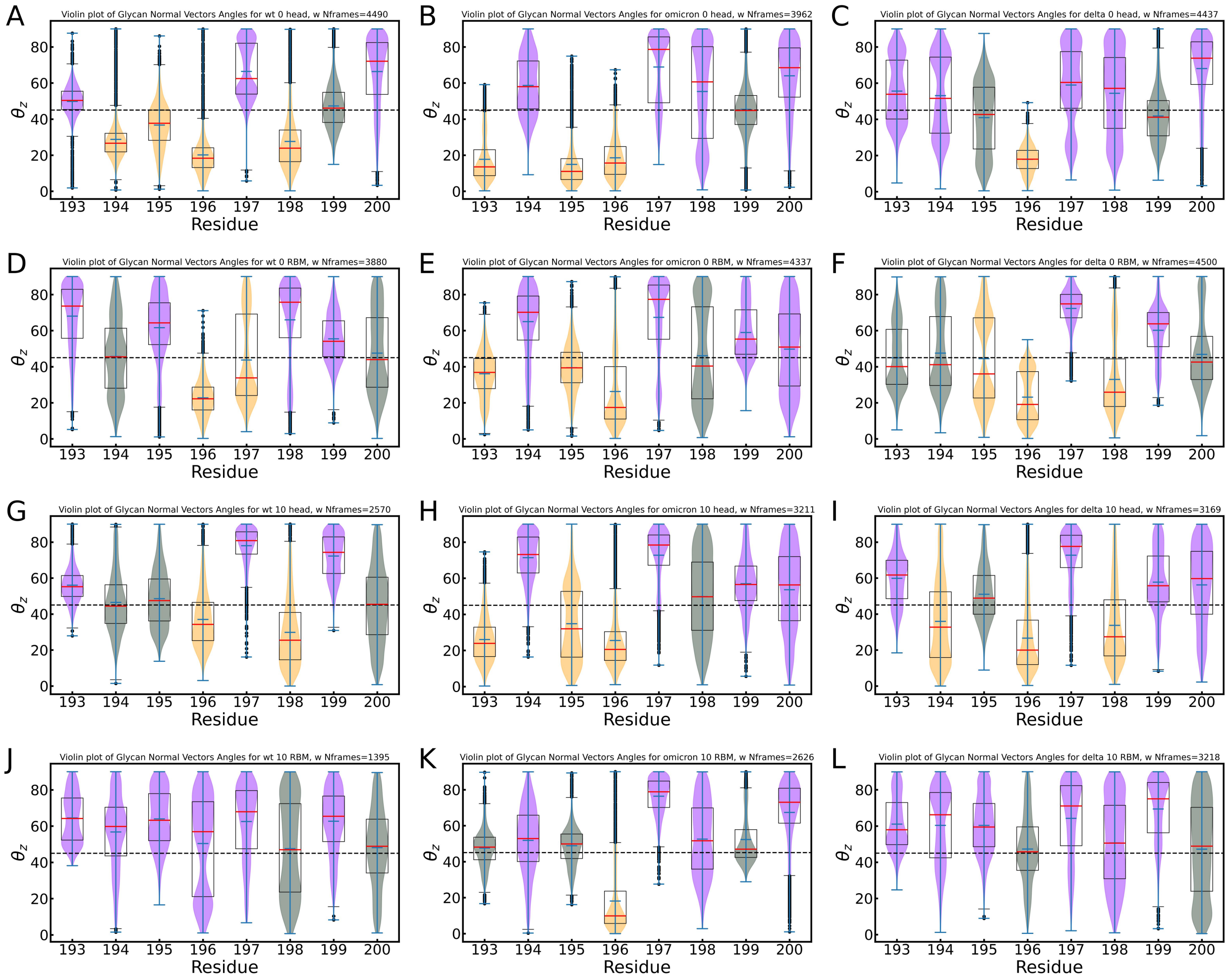

Figure S7: Violin and box plots of the angles (θ_z) of the sugar ring of the glycan with respect to the Z-axis (defined in Figure S6), 0 is parallel and 90 is perpendicular to the Z-axis. Red lines of the box indicate the median value, and the blue bars of the violin plot indicate the max, min, and mean value. Colors of the violin plots highlight median values higher than 50 (purple, approximately perpendicular), lower than 40 (pastel orange, approximately parallel) or in the threshold between 40 and 50 degrees (grey). As a reference, a segmented line at 45 degrees is added. Black dots are data considered outliers by the box plots. Box and violin plots were computed using the Matplotlib functions. Panels (A-F) are from simulations onto the hydrophobic surface, and (G-L) onto the hydrophilic surface for WT(left), Omicron(center), and Delta (right) variants. (A-C, G-I) correspond to simulations with the glycan within the head-RBD and the surface, and (D-F, J-L) with the glycan within the RBM and the surface.

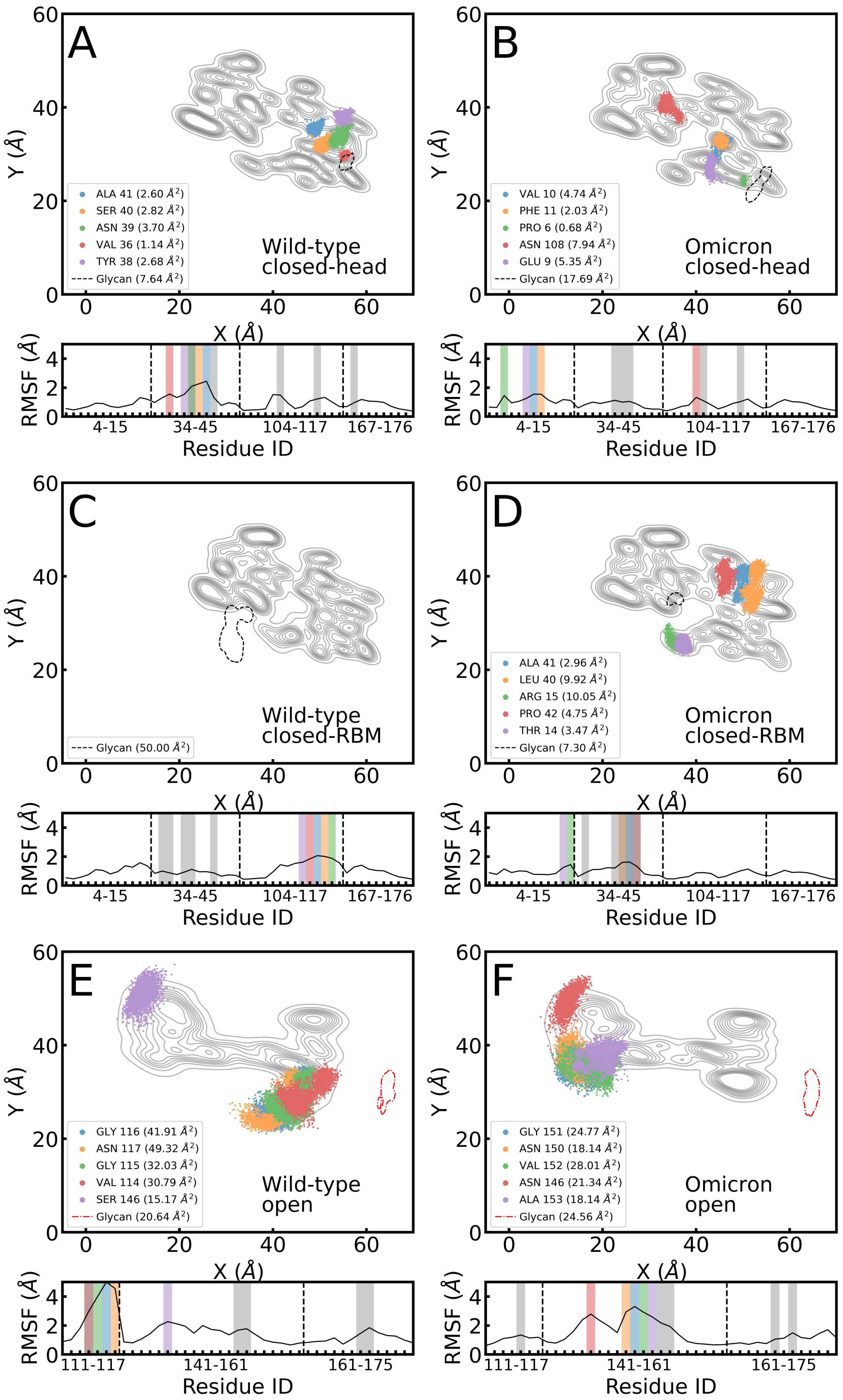

Figure S8: Footprint of the top five protein residues in the contact region with the highest RMSF values and area covered by glycan (dashed line) for the closed-RBD and open configurations onto the hydrophilic surface for WT (left column) and Omicron variants (right column). Panel ordering is consistent with Figures 5, 4, and S2. RMSF values for residues in the contact region are shown below each footprint, with colored regions highlighting the residues shown in the footprint and grey regions corresponding to residues with the highest number of surface contacts (Fig. S2). Red dot-dashed lines in (E) and (F) are a reminder that the glycan in the open conformations is not adsorbed (normal distance > 14 Å).

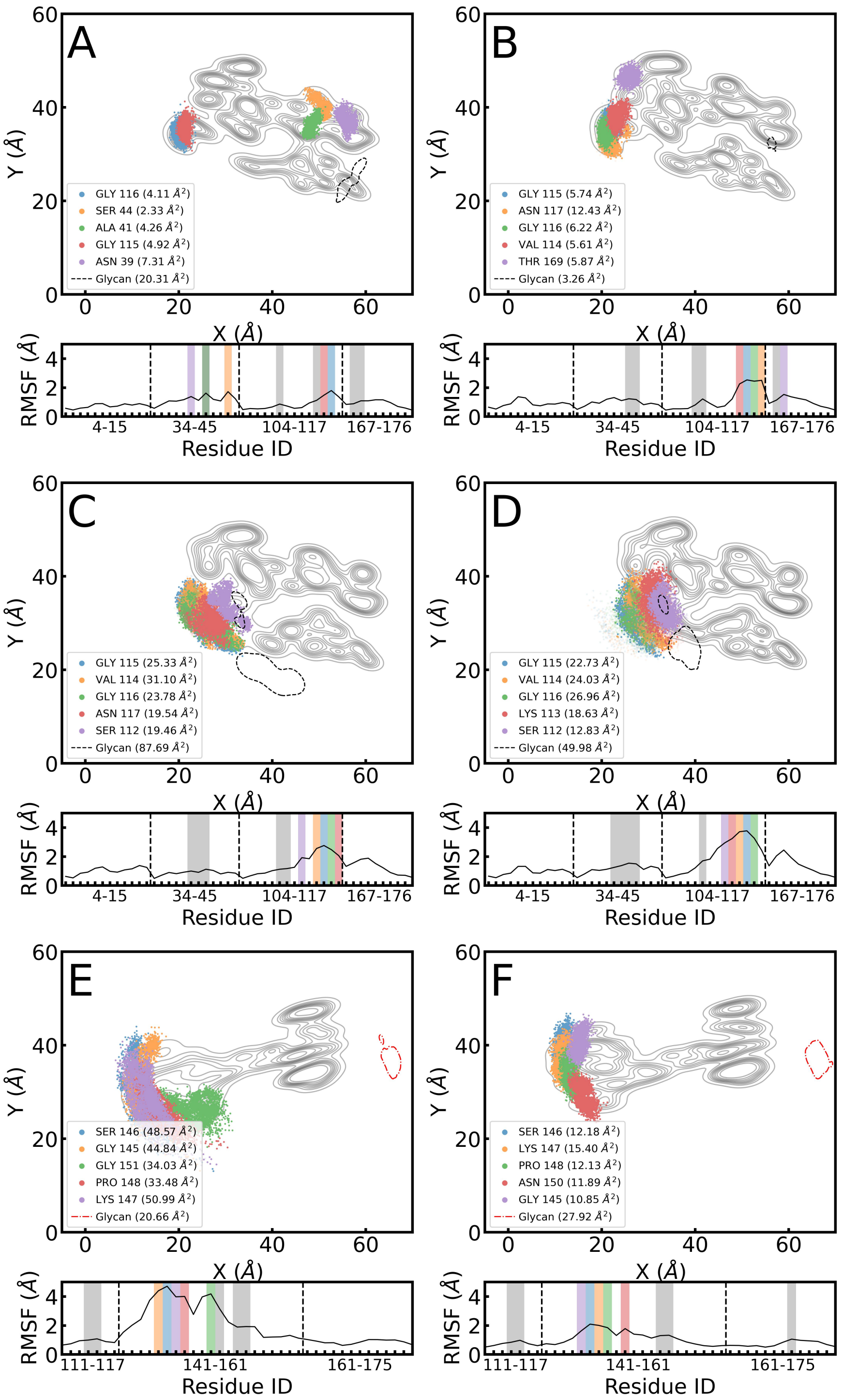

Figure S9: Footprint of the top five protein residues in the contact region with the highest RMSF values and area covered by glycan (dashed line) for the closed-RBD and open configurations the hydrophobic (left column) and hydrophilic (right column) surfaces for the closed- and open-RBD configurations for Delta variant. Panel ordering is consistent with Figure S3. RMSF values for residues in the contact region are shown below each footprint, with colored regions highlighting the residues shown in the footprint and grey regions corresponding to residues with the highest number of surface contacts (Fig. S3). Red dot-dashed lines in (E) and (F) are a reminder that the glycan in the open conformations is not adsorbed (normal distance > 14 Å).

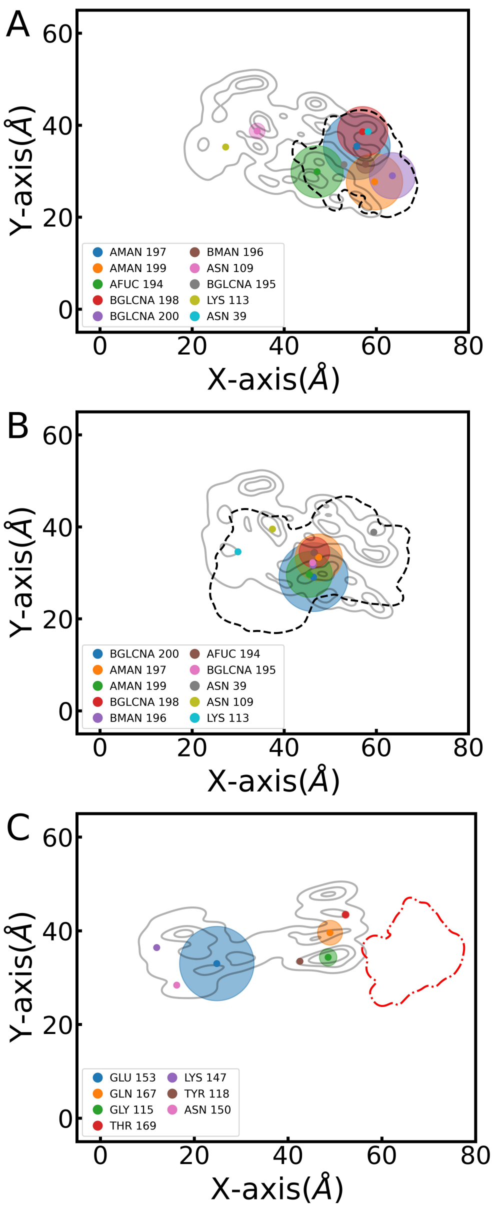

Figure S10: Hydrogen bonds occurrence between the protein and the surface during the simulation time (shown as %). H-bonds are represented in circles centered at the center-of-mass of each residue with proportional size to the percentage of H-bonding during the trajectory. The three contour levels in gray represent the mean positions of the protein, and the single contour in segment-black indicates the mean position of the glycan. Panels display results for (A) closed-RBD with the glycan between the RBD head and the surface, and (B) closed-RBD with the glycan between the RBM and the surface, and (C) open-RBD configuration.

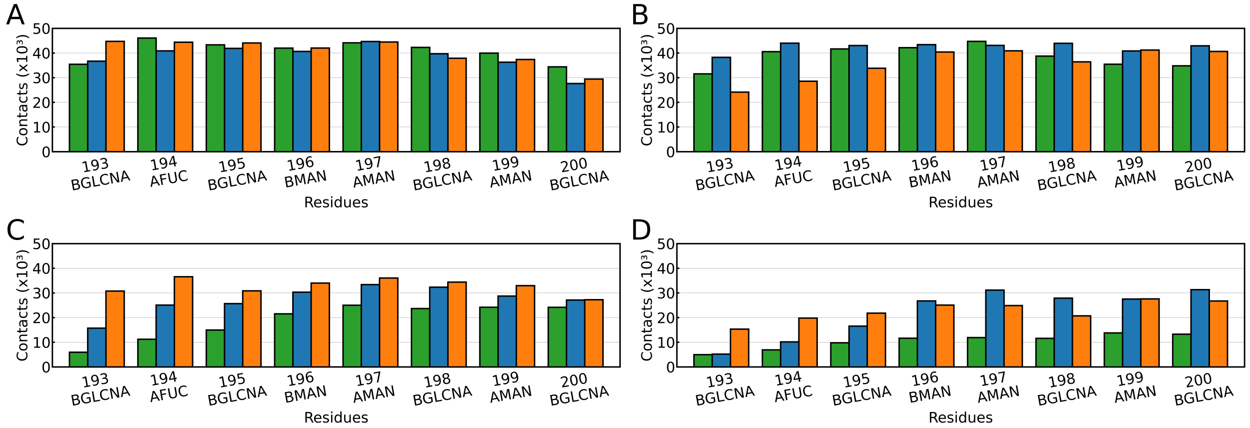

Figure S11: Glycan contacts with the surface when positioned between the surface and (A, C) the RBD head, or (B, D) the RBM region. Panels (A) and (B) correspond to a hydrophobic surface, while (C) and (D) correspond to a hydrophilic surface. Results are shown for the three VoCs; WT (green), delta (blue), and Omicron (orange).

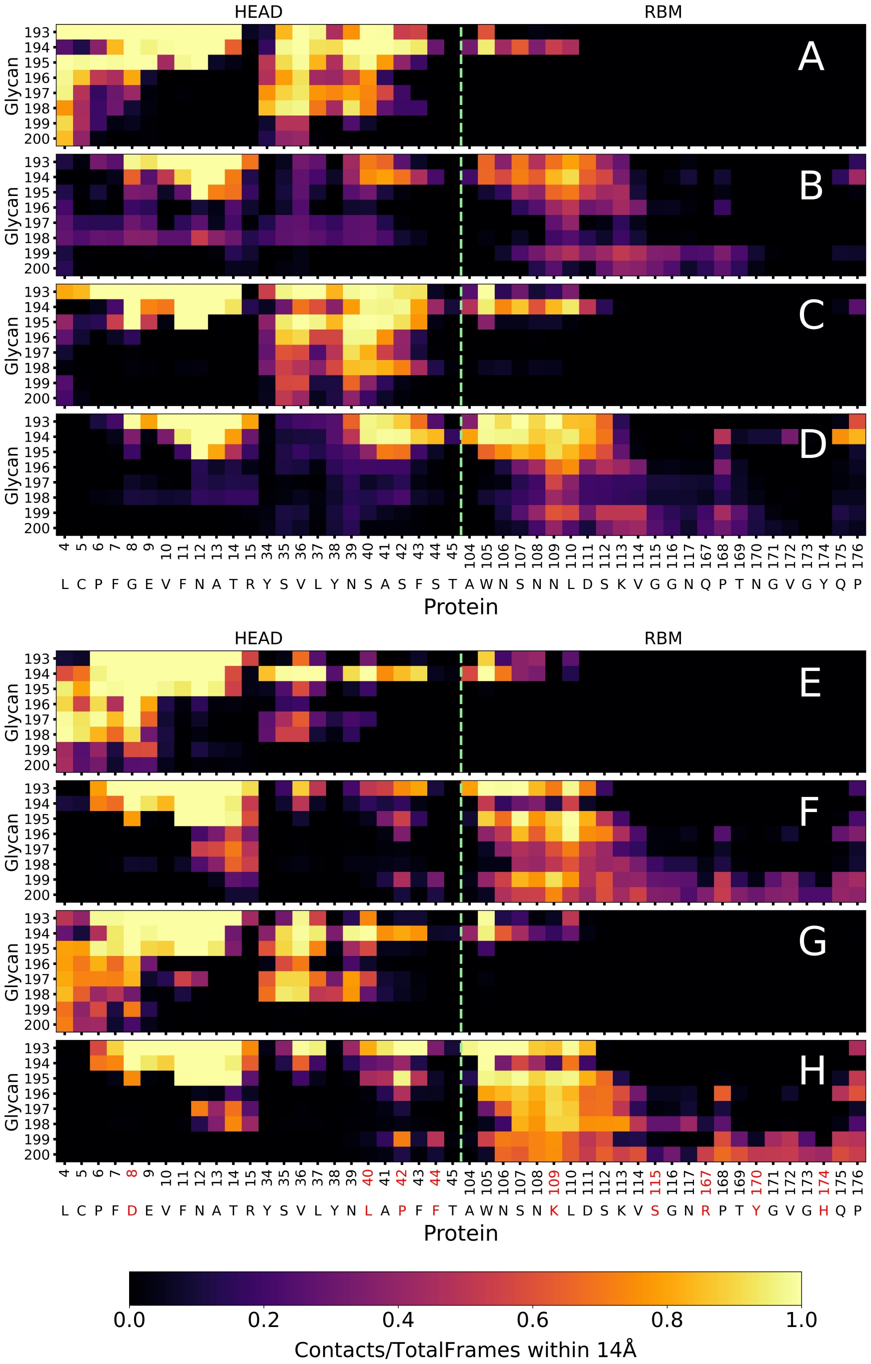

Figure S12: Contact maps between glycan and protein residues within the contact region for (A–D) WT and (E–H) Omicron. Panels (A, B, E, F) show results from simulations with a hydrophobic surface, while (C, D, G, H) correspond to a hydrophilic surface. In panels (A, C, E, G), the glycan is positioned between the RBD head and the surface; in (B, D, F, H), it is located between the receptor-binding motif (RBM) and the surface. A horizontal segmented lime green line in each map indicates the boundary separating residues in the RBD head from those in the RBM region of the RBD. Contacts are considered as residue-residue distances under 14Å.

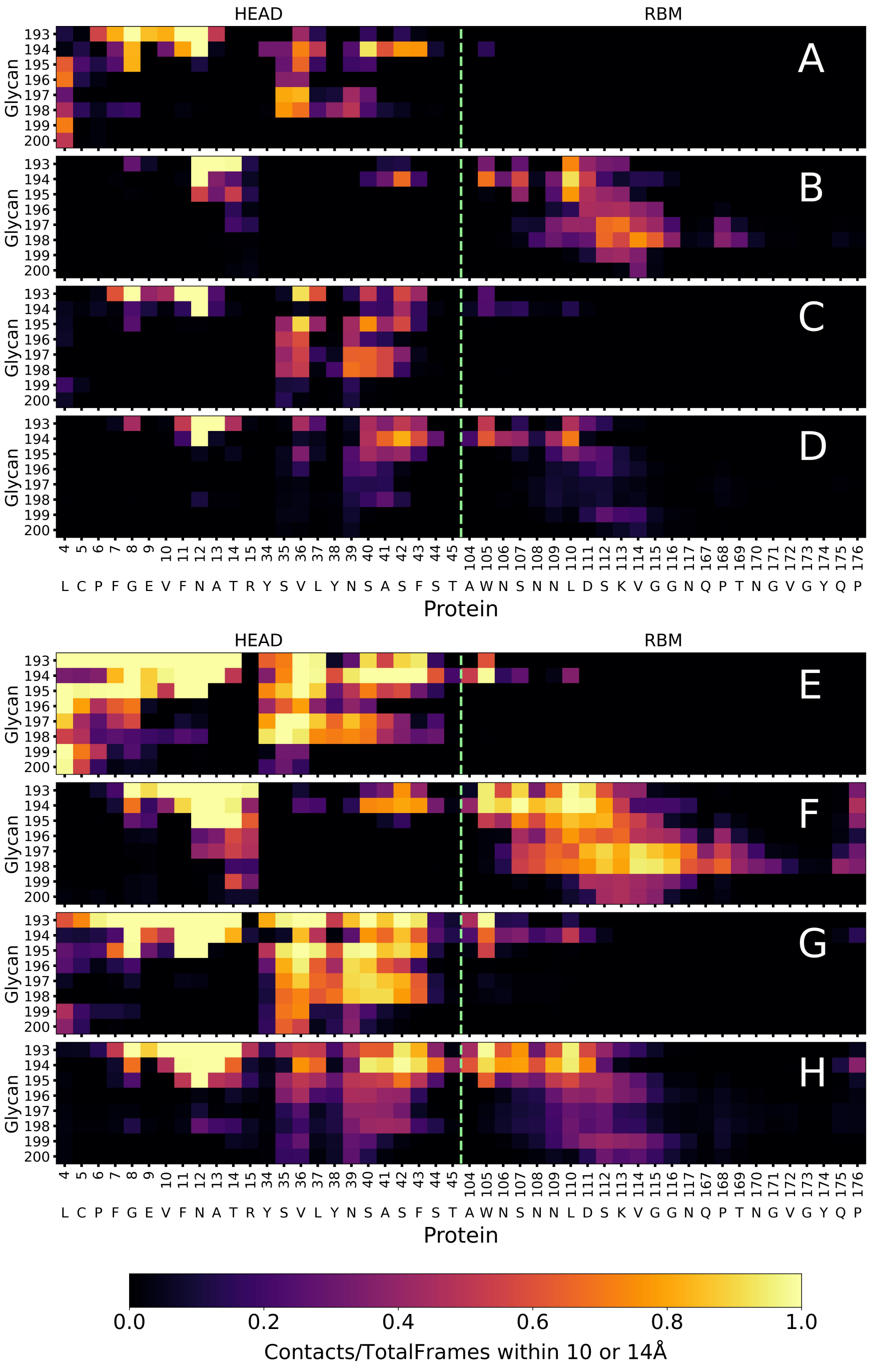

Figure S13: Contact maps between glycan and protein residues within the contact region for the Delta variant. A horizontal segmented lime green line in each map indicates the boundary separating residues in the RBD head from those in the RBM region of the RBD. Contacts are considered as residue-residue distances under (A-D) 10Å and (E-H) 14Å. Analogue to other figures with contact maps, panels (A, B, E, F) show results from simulations with a hydrophobic surface, while (C, D, G, H) correspond to a hydrophilic surface. In panels (A, C, E, G), the glycan is positioned between the RBD head and the surface; in (B, D, F, H), it is located between the receptor-binding motif (RBM) and the surface.

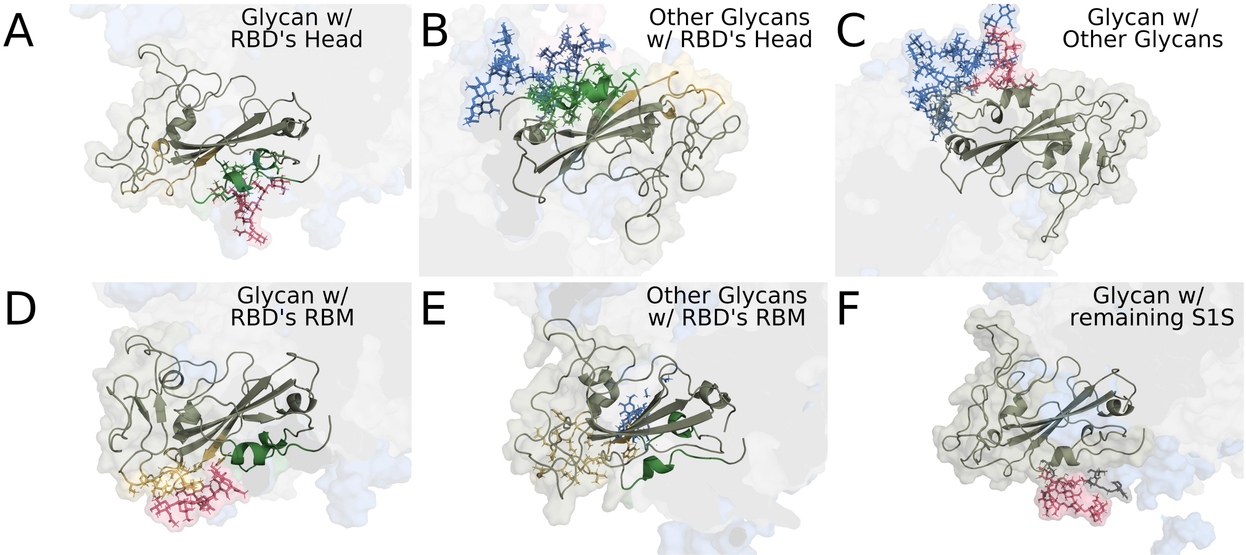

Figure S14: Snapshots showing the context of different regions of the RBD+glycan complex with the remaining Spike ectodomain glycoprotein for open RBDs of WT. Glycan contacts with (A) the head and (D) the RBM of its RBD; other non-RBD glycans of the Spike with (B) the head and (E) the RBM; glycan with (C) other non-RBD glycans and with (F) protein residues of the remaining Spike ectodomain (SpEct) glycoprotein. In the Figure, the RBD head is in green, the RBM region of the RBD is in orange, the glycan is in red, and the remaining SpEct protein is in grey, and the remaining glycans of the SpEct are in blue. Residues in sticks are at 7Å distance from the reference region. Reference regions : (A,B,D,E) Glycan of the RBDs, (C) head of RBD, and (F) RBM of the RBD.

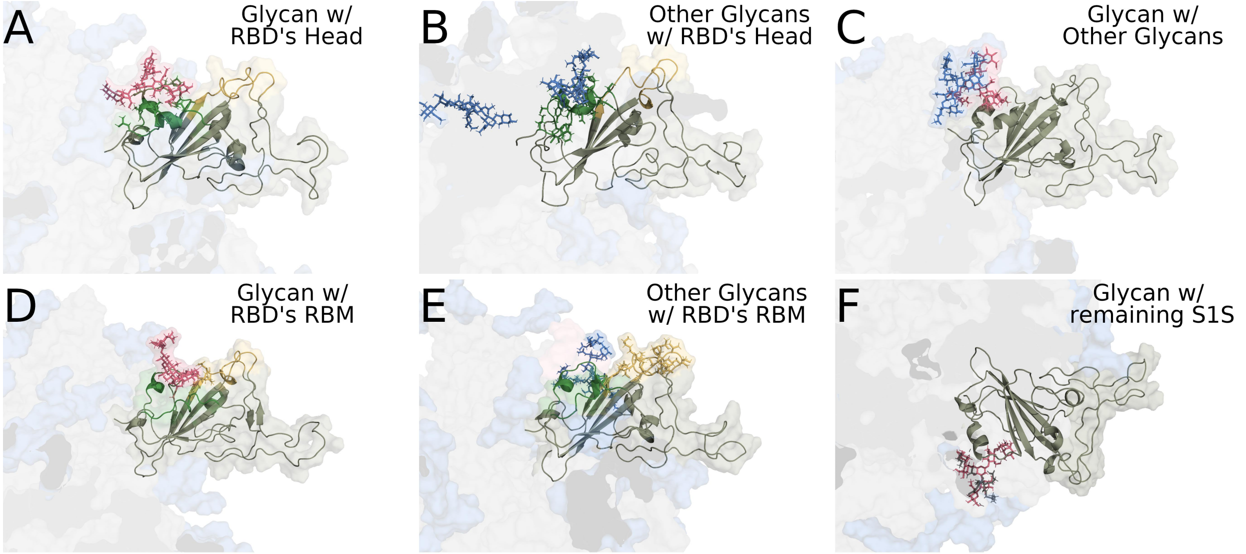

Figure S15: Snapshots showing the context of different regions of the RBD+glycan complex with the remaining Spike ectodomain glycoprotein for open RBDs of Omicron. Panel display and color designation are the same as Figure S14.

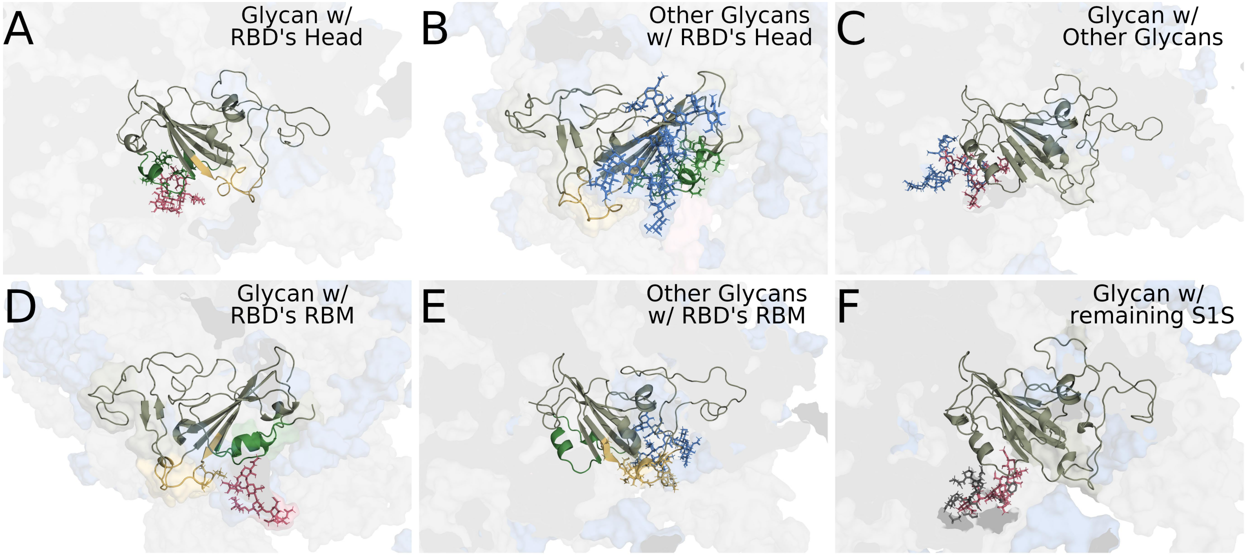

Figure S16: Snapshots showing the context of different regions of the RBD+glycan complex with the remaining Spike ectodomain glycoprotein for closed RBDs of WT. Panel display and color designation are the same as Figures S14 and S15.

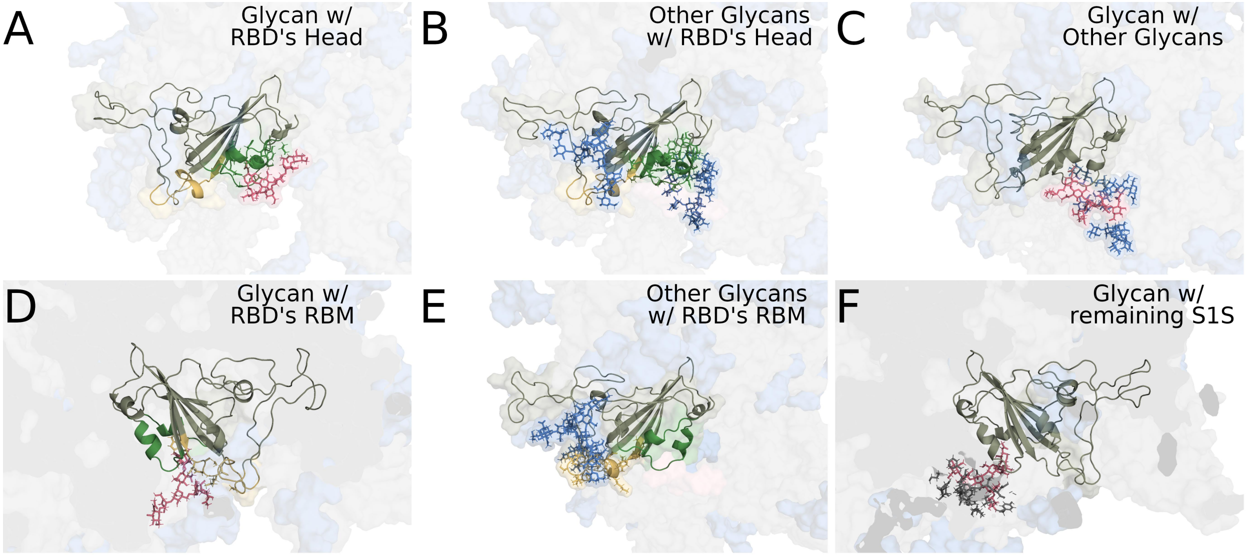

Figure S17: Snapshots showing the context of different regions of the RBD+glycan complex with the remaining Spike ectodomain glycoprotein for closed RBDs of Omicron. Panel display and color designation are the same as Figures S14, S15, and S16.

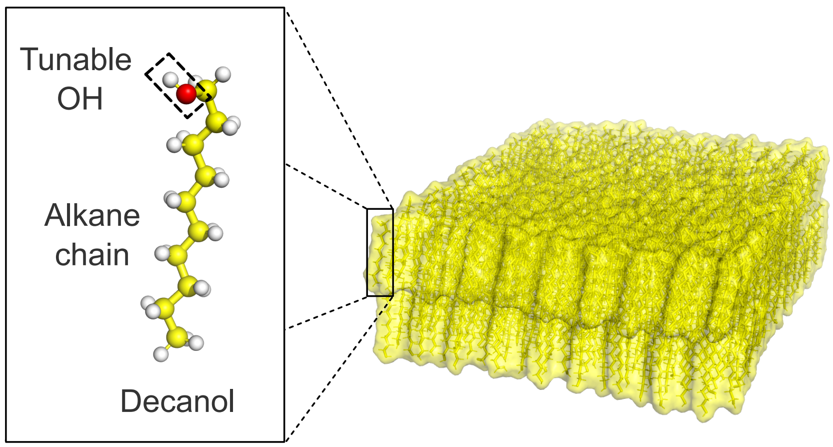

Figure S18: Graphical representation of the polarizable bilayer, indicating the chemistry of the model at each decanol molecule in the PBL.

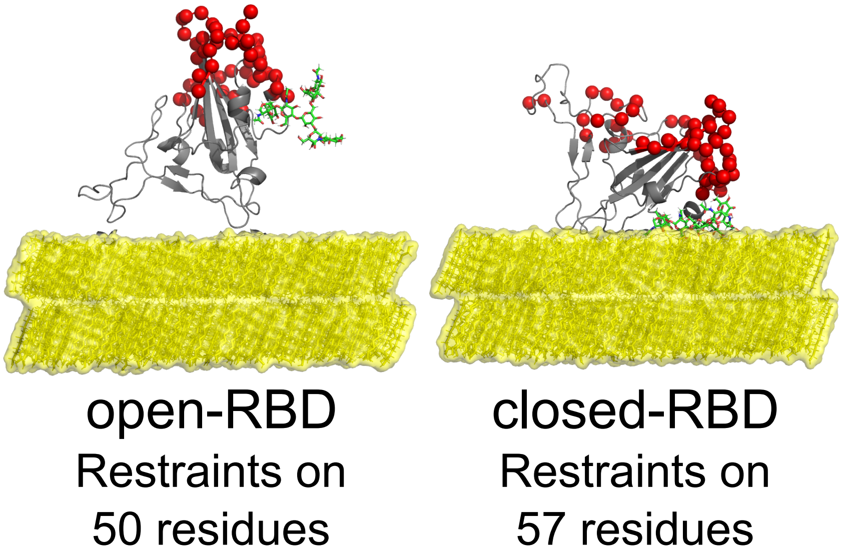

Figure S19: Graphical representation of the RBD glycoprotein region in the presence of the PBL, highlighting the position of the restraints used in our previous work for open-RBDs (left), and in our current work for the closed-RBDs (right).

References

(1) Milchev, A.; Binder, K. How does stiffness of polymer chains affect their adsorption transition? *The Journal of chemical physics* **2020**, *152*.

(2) Bosch, A. M.; Guzman, H. V.; Pérez, R. Adsorption-Driven Deformation and Footprints of the RBD Proteins in SARS-CoV-2 Variants on Biological and Inanimate Surfaces. *Journal of Chemical Information and Modeling* **2024**, *64*, 5977–5990.
